# Modeling the spatial distribution of grazing intensity in Kazakhstan

**DOI:** 10.1371/journal.pone.0210051

**Published:** 2019-01-11

**Authors:** Brett R. Hankerson, Florian Schierhorn, Alexander V. Prishchepov, Changxing Dong, Christina Eisfelder, Daniel Müller

**Affiliations:** 1 Leibniz Institute of Agricultural Development in Transition Economies (IAMO), Halle (Saale), Germany; 2 Geography Department, Humboldt-Universität zu Berlin, Berlin, Germany; 3 Department of Geosciences and Natural Resource Management (IGN), University of Copenhagen, Copenhagen, Denmark; 4 Institute of Environmental Sciences, Kazan Federal University, Kazan, Russia; 5 German Remote Sensing Data Center (DFD), German Aerospace Center (DLR), Oberpfaffenhofen, Germany; 6 Integrative Research Institute on Transformations of Human-Environment Systems (IRI THESys), Humboldt-Universität zu Berlin, Berlin, Germany; West African Science Service Centre on Climate Change and Adapted Land Use/Research Department, BURKINA FASO

## Abstract

With increasing affluence in many developing countries, the demand for livestock products is rising and the increasing feed requirement contributes to pressure on land resources for food and energy production. However, there is currently a knowledge gap in our ability to assess the extent and intensity of the utilization of land by livestock, which is the single largest land use in the world. We developed a spatial model that combines fine-scale livestock numbers with their associated energy requirements to distribute livestock grazing demand onto a map of energy supply, with the aim of estimating where and to what degree pasture is being utilized. We applied our model to Kazakhstan, which contains large grassland areas that historically have been used for extensive livestock production but for which the current extent, and thus the potential for increasing livestock production, is unknown. We measured the grazing demand of Kazakh livestock in 2015 at 286 Petajoules, which was 25% of the estimated maximum sustainable energy supply that is available to livestock for grazing. The model resulted in a grazed area of 1.22 million km^2^, or 48% of the area theoretically available for grazing in Kazakhstan, with most utilized land grazed at low intensities (average off-take rate was 13% of total biomass energy production). Under a conservative scenario, our estimations showed a production potential of 0.13 million tons of beef additional to 2015 production (31% increase), and much more with utilization of distant pastures. This model is an important step forward in evaluating pasture use and available land resources, and can be adapted at any spatial scale for any region in the world.

## Introduction

The global livestock sector has an estimated value of upwards of $1.4 trillion and employs more than 1.3 billion people [1]. Animal products make up 40% of the current global food demand [2], which is expected to double by 2050 (from 2005 levels) [3]. The largest increases in the demand for livestock products are expected to occur in developing and transitioning countries [4]. Livestock production already accounts for around 75% of all agricultural land use, including land that is classified as pasture and cropland that is dedicated to feed production [5]. The livestock sector is also responsible for up to 18% of all anthropogenic greenhouse gas emissions, due in large part to methane emission during enteric fermentation in ruminants, as well as emissions of nitrous oxide from nitrogen-based fertilizers, methane from manure management, and carbon related to land use change [6]. Grazing patterns of livestock also play a huge role in native species abundance and distribution [7]. Yet, despite the large land requirement and significant environmental footprint of livestock production, knowledge gaps exist when it comes to the area, intensity, and spatial distribution of land that is devoted to livestock production.

Determining the land-use footprint of livestock is difficult. So far, the primary focus has been on land used for crops, due partially to the fact that it is easier to detect accurately with remote sensing: fields have distinguishable spectral characteristics, defined edges, and the location of fields is path dependent [8, 9]. Unlike cropland, pasture often consists of natural grassland, and differentiating grassland and pasture using remote detection is an arduous task that as of yet has no universal solution [10]. For this research, we define grassland as a biophysical land cover that includes areas with low-standing vegetation, including shrublands [11]. Pasture is defined as a land use that includes any area actively utilized by livestock for grazing, which can include grassland as well as other types of land cover, including cropland [12]. Pasture edges are often undefined, especially in areas with communal grazing, nomadic transhumance, and unclear or unenforced land ownership rights [13]. Consequently, little knowledge currently exists on the utilization of grasslands for pasture. Unfortunately, data on pasture extent are scarce, which hinders the accurate estimation of utilized and available land resources.

Several products attempt to map land use on a global scale. Erb et al. [14] and IIASA and FAO [15] utilized a subtractive approach (with differing classifications and definitions), while Ramankutty et al. [16] and Klein Goldewijk et al. [17] used different allocation rules that combine remote sensing-derived land cover with national statistics. These four global products share a modicum of agreement (17% of the land use classification is in full agreement, with an additional 30% where three maps agree) but marginal pasture areas are particularly discrepant, such as in semi-arid and tundra regions, which are sparsely vegetated and contain transitional classes [12]. Similarly, global remote sensing products often have lower classification accuracies for grassland ecosystems, particularly in areas where transition classes and ecotones are common [18–20]. Thus, estimating pasture area with global land cover products can only be used as general approximations. As livestock enumeration using remote sensing is currently impractical, it is necessary to develop methods of pasture allocation that combine remote sensing with ground-based data and information on local grazing practices, which is crucial for modeling livestock distribution.

The Gridded Livestock of the World (GLW) was the first global, gridded map of livestock density [21]. With finer resolution (1 km) and more up-to-date statistics, the GLW was improved in 2014 [22], and again in 2018 with a more advanced spatial weighting model to allocate livestock [23]. The authors used the suitability for pasture as well as socioeconomic factors to predict the density of different livestock species, using national and sub-national livestock statistics. However, the accuracies of the GLW are lowest in semi-arid regions and generally in under-populated areas where livestock numbers are often underrepresented due to seasonal movement of stock in remote and inaccessible areas, and information on specific climate, biophysical and socioeconomic characteristics are not available [21]. Additionally, despite the high resolution, for many countries livestock statistics were used from first-level administrative divisions. For such countries, including Russia, Kazakhstan, Algeria, Saudi Arabia, Indonesia, and Sudan (all among the world’s 15 largest countries), this severely limits the suitability of the GLW for regional and local studies [22]. There have also been attempts to assess fine-scale livestock dynamics with spatial extrapolation of local livestock numbers as a proxy for the area used as pasture [24]. However, such approaches fail to account for the productivity of grasslands, the biomass intake by the different types of grazing livestock, or regional grazing practices.

We developed a model to estimate the grazing intensity of livestock (cattle, sheep, goats, and horses) in Kazakhstan. Grazing intensity is the percent utilization of net primary production, and allows for the gridded estimation of grazing pressure based on natural productivity. Kazakhstan is particularly interesting because of its long history of livestock rearing and the vast land resources suitable for grazing. The ninth-largest country in the world, Kazakhstan’s permanent pastures and meadows were estimated at 1.87 million km^2^ (69% of total land area) in 2014 by the Food and Agriculture Organization (FAO); by comparison, Russia had 0.93 million km^2^ and the United States 2.51 million km^2^ [25]. Historically, large numbers of wild herbivores shared Kazakhstan’s grasslands with livestock, the most populous being the saiga antelope (*Saiga tatarica*). However, during the 1990s, populations of saiga plummeted due to the loss of enforcement of hunting quotas and illegal trade in saiga horns [26, 27]. Saiga are also vulnerable to periodic, catastrophic die-offs, one of which occurred in spring 2015 [28, 29]. In summer 2015, saiga in Kazakhstan numbered around 84,000 [30]. Other wild herbivores of note in Kazakhstan include the kulan (*Equus hemionus*) and the goitered gazelle (*Gazella subgutturosa*), which in 2015 numbered around 3,500 and 13,000, respectively [30]. These numbers pale in comparison to grazing livestock numbers (6.2 million cattle, 18 million sheep and goats, and 2.1 million horses in 2015) [31]. Similarly, domestic camels in 2015 numbered 170,000 [31]. While in some districts they represented a noticeable source of grazing demand, camels and wild herbivores contributed very little to total grazing intensity at the national scale and thus were not considered in this research.

The model estimates the distribution of livestock grazing and contributes to closing the knowledge gap regarding the spatial dimension and intensity of pasture use. We used statistical data on livestock numbers and fodder production along with recommended energy intake levels per animal type to estimate the livestock grazing demand (the total energy consumed through grazing) for Kazakhstan in 2015. Based on reported grazing practices, we applied this demand to biomass productivity maps to determine the most likely distribution of utilized pasture. The inverse of the result is the amount of energy that is available for grazing, but not currently utilized. Specifically, we were interested in whether the differences in grazing practices among the predominant farm structures and climatic zones would be captured in the output. We aimed to answer the following research questions:

1. What was the energy demand and associated pasture requirement for Kazakh grazing livestock in 2015?
2. What was the spatial distribution of this pasture?
3. Where was the pasture being underutilized, and what is the potential for increasing the herd size and the production of meat and milk?

## Materials and methods

### Study area

Situated in the middle of the Eurasian Steppe, Kazakhstan has a northeast-southwest precipitation gradient that allows rain-fed grain production in the north (upwards of 350 mm/year), but transitions quickly to dry steppe and further to desert climate in the southwest (less than 100 mm/year). Precipitation does not suffice for cropland agriculture in many parts of the country where grazing is the only agriculturally significant activity possible [11].

Unlike many other grassland regions of the world, Kazakhstan once supported a much larger livestock population than it has at present, particularly while a part of the Soviet Union, when livestock production was viewed as a pillar of centrally planned economies [32]. After the Soviet Union dissolved, livestock numbers in Kazakhstan plummeted and widespread abandonment of pastures and croplands occurred [24, 33, 34]. While livestock numbers have been steadily recovering since 2000, as of 2015 they are still only two-thirds of 1990 levels [31].

However, Soviet levels of production cannot be assumed the desirable goal, as it is generally accepted (though poorly documented) that soil degradation was widespread, with overgrazing a major contributor [35]. Furthermore, degradation of grazing lands has continued up to the present, despite the drastic reduction in grazing livestock numbers [36]. Under the Soviet system, the human population was settled in permanent villages. Livestock migration continued, but was structured and strictly regulated. To supplement grazing (especially during winter), there was a large, subsidized network of fodder supply. When the Soviet Union dissolved, so too did the fodder supply network. Yet the human population remained sedentary, with livestock held almost exclusively in small, household farms. This led to localized overgrazing around settlements, as distant outposts fell into disrepair, and long-distance migration all but stopped [37].

### Kazakh farm structure

The type of farm that livestock are raised on in Kazakhstan determines to a great degree how the livestock are grazed [32]. There are three distinct farm types in Kazakhstan: the largest farms are called agricultural enterprises (AE), ranging from a few thousand to tens of thousands of animals. These farms are the spiritual (and often actual) descendants of Soviet farms and usually have the resources to provide high-quality fodder and support longer-distance grazing migrations. The second group are the private farms (PF). Since 2000, this is the fastest-growing farm type, and the most diverse in terms of size and grazing practices, ranging from tens to thousands of animals. The third and smallest farm type, households (HH), typically have only a few animals per unit, but overall in Kazakhstan, 60% of grazing livestock are kept in this farm type. Livestock are usually housed overnight in the owner’s backyard, meaning that their grazing area is limited to the distance they can walk in one half-day. This immobility results from the sedentarization of farms under the Soviet regime, and the path dependency following its dissolution [38–40]. Prior to the Soviet era, livestock generally grazed in large, transhumant, communal herds [41].

### Model development

**Fig 1:**
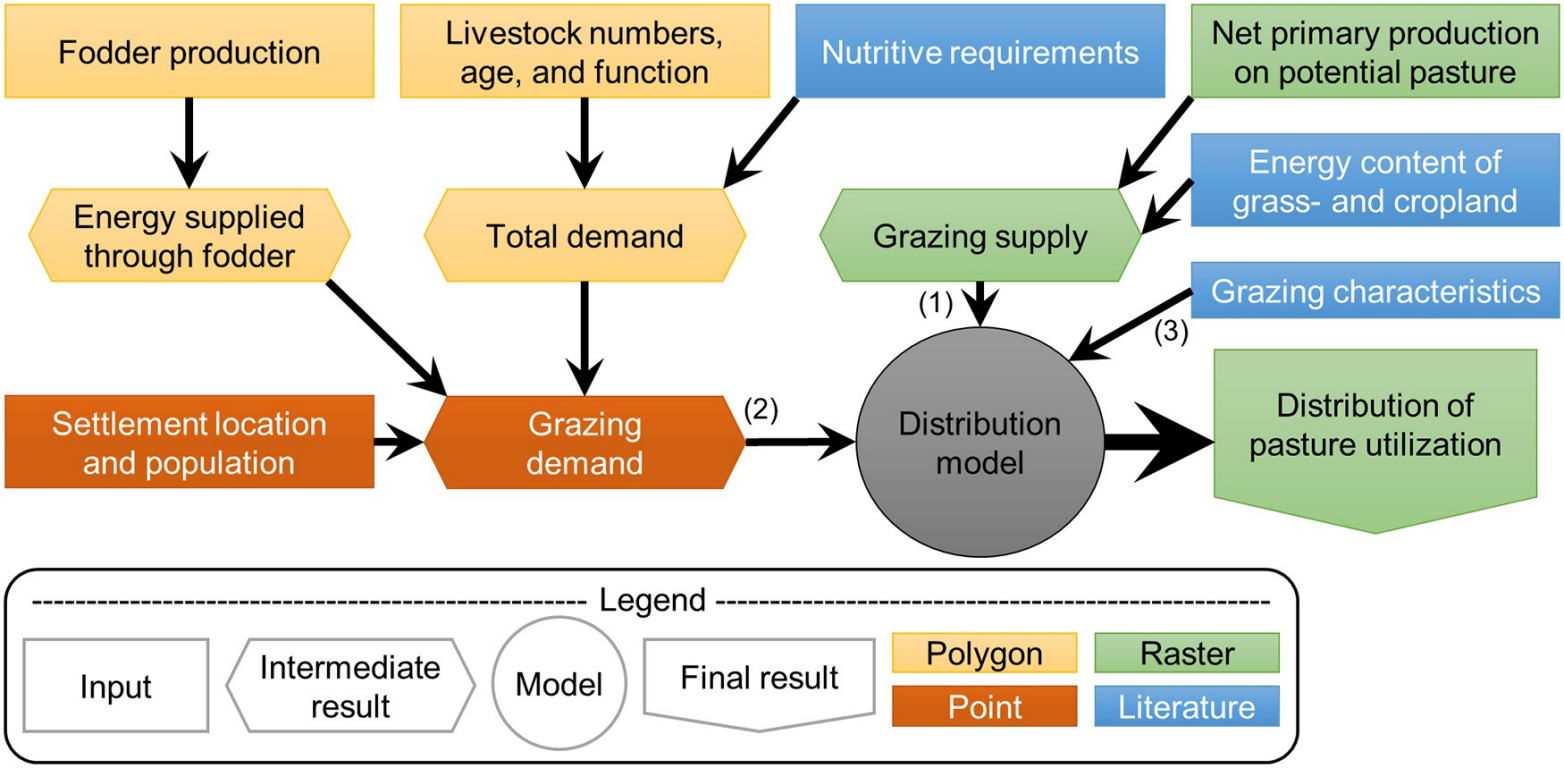
Flow chart for the distribution of utilized pasture. (1), (2), and (3) are the inputs to the distribution model. Polygon data is at the district level, point data is at the settlement level (there are 7,234 settlements in Kazakhstan), and raster data is resolved to 500 meters. Nutritive requirements and grazing characteristics are derived from literature and defined differently for the three farm types.

Three main components are used as inputs to our livestock distribution model (Fig 1). Grazing supply (1) is the base map, which consists of energy stored in all the biomass that could be used for grazing. Grazing demand (2) is the sum of all energy required by livestock for growth, maintenance, and lactation, less the amount of energy that they receive through supplied fodder. Energy supplied through fodder is calculated based on statistics [31] and energy conversion ratios used in Kazakhstan [42]. To spatially distribute the grazing demand, grazing characteristics (3), including home base location, daily grazing range, seasonal migration, and off-take rate (percentage of produced biomass that is consumed) need to be defined.

Knowing that the livestock are clustered around settlements [43], and assuming that they, to some extent, behave as optimal foragers [44, 45], we defined a search algorithm to seek out the most productive grassland for grazing surrounding the geographic coordinates of the settlements [46]. Grassland productivity was captured using net primary productivity (NPP) [47, 48]. As a way to disaggregate livestock numbers from district-level statistics to the settlement level, the human population of each settlement was used [49], along with a piecewise linear function (S1 Equation) describing the relationship between settlement size and number of livestock owners, determined from expert opinion and personal observation (S1 Fig).

The search radius defines the area around the settlement within which pixels are selected. Our model allows the search radii to be defined independently for each livestock/farm type combination. The search radii used in this research were developed based on farmer interviews and the findings of Kamp et al. [50] and Coughenour et al. [43]. Three different radii were used to simulate three different grazing practices found in Kazakhstan. For cattle, sheep, and goats on households and private farms, the search radius was set to 2 km to represent the relative immobility of these livestock. For cattle, sheep, and goats on agricultural enterprises, the search radius was set to 5 km, to represent the increased resources of the enterprises to seek better pastures farther from the settlement (displaying slightly more “optimal forager” activity) [44]. For horses on all farm types, the search radius was set to 10 km, because horses are more mobile and rarely return to the settlement overnight, and thus have the greatest ability to approach optimal foraging. For a summary of the assumptions made in the distribution model, and their implications, see S1 Table. To distribute the demand spatially and realistically, we use an iterative approach. For each combination of livestock type and farm type, a radius r is defined (2, 5, or 10 km, as defined above). For every settlement, the pixels within r are sorted according to their NPP value, and the settlement’s demand is distributed to the pixels, choosing higher values first, and precluding chosen pixels from possible duplicate distribution. If the demand is not met within the initial search radius, the search radius is increased to 2r. The distribution process repeats with increasing search radii (3r, 4r, etc.) until the demand is satisfied. Moreover, the distribution for all settlements is performed simultaneously to allow fair competition, reflecting the reality. This results in the distribution of all available land in (n-1) radii around a settlement, with the final radius containing the last pixels distributed to meet the demand. A magnification of the results, showing the settlement-level pixel distribution, is shown in S2 Fig. We chose to distribute the demand of sheep and goats before cattle, though their grazing areas for the most part overlap (literature indicates cattle will travel slightly further during a day) [51, 52]. Thus, for sheep, goats, and cattle, farm type is more important than animal type, and the algorithm distributed all sheep, goats, and cattle in households first, then private farms, and finally agricultural enterprises. Horses, on the other hand, typically graze separate from the other livestock, and occupy areas distant from settlements, because they are more mobile and remain on the pasture overnight. To account for this, horses of all farm types were distributed last, thus receiving the pasture furthest from the settlements. Based on the characteristics of the different farm types, and the mobility of the different animal types, the livestock were distributed in this order: 1. sheep and goats in households, 2. cattle in households, 3. sheep and goats in private farms, 4. cattle in private farms, 5. sheep and goats in agricultural enterprises, 6. cattle in agricultural enterprises, 7. horses in households, 8. horses in private farms, and 9. horses in agricultural enterprises. The model is run within the R environment, with image processing carried out in R and in QGIS [53, 54].

### Grazing supply

For grazing supply, we used net primary productivity (NPP) produced by Eisfelder et al. [47] who used the Biosphere Energy Transfer Hydrology Model (BETHY/DLR) [55]. NPP is dry matter production per unit area per unit time. We used the best available land-cover map for the region [11] to mask out areas that are unavailable for grazing, namely forest, water, ice, and artificial surfaces. Grazing is known to occur in all types of protected areas except nature preserves (*zapovedniki*), which cover 1370 km^2^ and have the highest level of protection [56]. Thus, nature preserves were also excluded.

**Fig 2:**
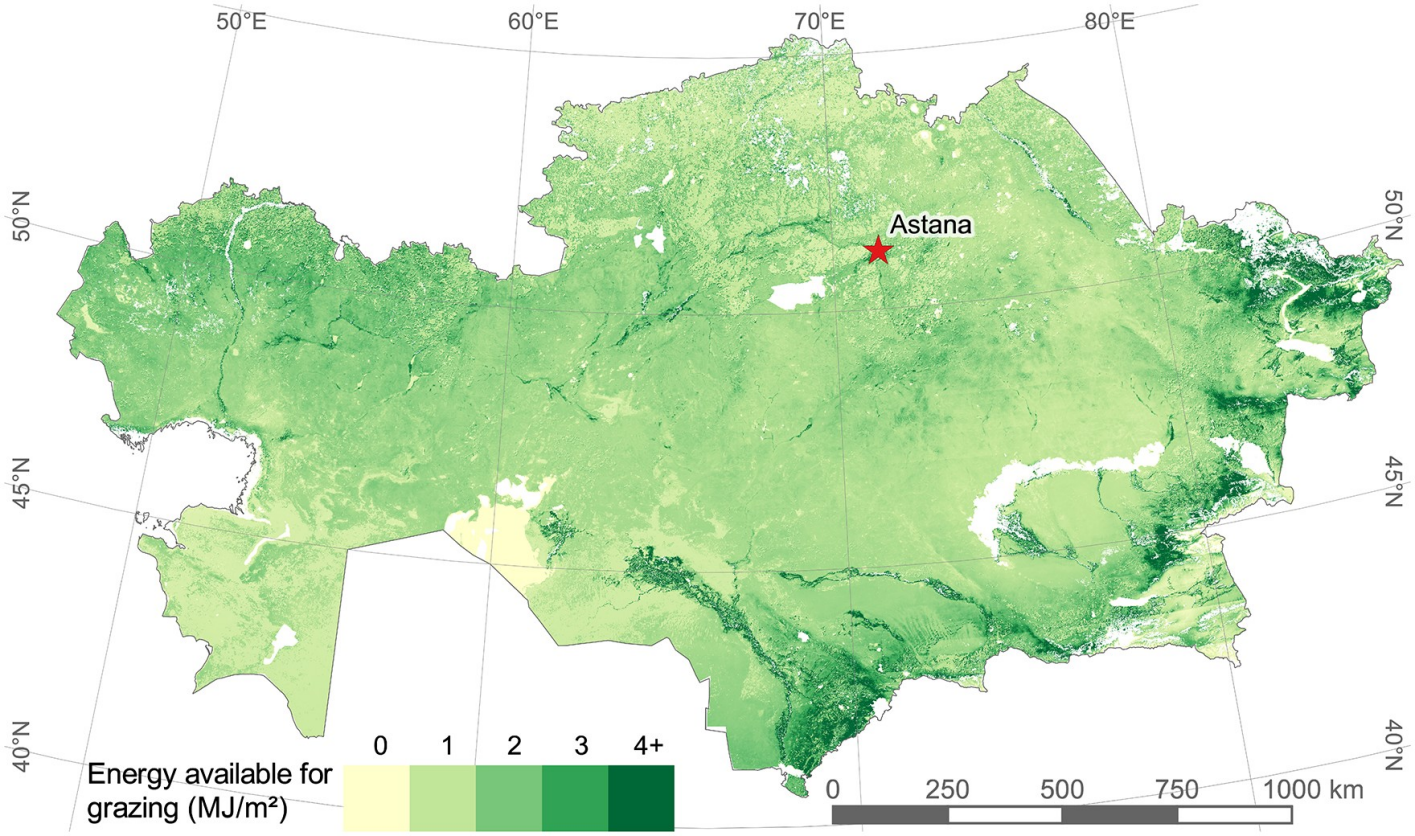
Map of annual available grazing supply (MJ/m^2^) derived in this study. Based on total NPP measured by Eisfelder et al. [47]. The land-cover classification used for masking is from Klein et al. [11], and the protected area mask is from Kamp et al. [56]. Low available NPP for foraging on croplands can easily be seen in the northcentral. White areas are unavailable for grazing.

Areas defined as “bare soils” were included as grassland because they, though low in NPP, contain areas where grazing is known to occur [43]. A common practice in Kazakhstan is the foraging of crop residues after harvest, especially on wheat fields. To account for this, we split the map into a grassland and a cropland map. For both maps, we created a composite with the mean of the nine-year NPP data (compiled into annual totals) and converted NPP (grams carbon/square meter) to biomass production (grams dry matter/square meter) using the conversion coefficient of 0.47 grams carbon/grams dry matter [57]. On the grassland map, we converted biomass to available energy using a value of 8.6 Megajoules (MJ) per kilogram dry matter (kgDM), based on literature from similar regions and climates (Fig 1) [58–60]. The total energy available from grasslands was calculated to be 3537 PJ. To estimate the biomass available from the foraging of crop residues, we applied a harvest index of 0.48 (using wheat as the base reference) to the annual NPP [61, 62]. The harvest index is the mass ratio of crop yield (grain) to the crop’s total aboveground biomass [63]. Wheat is the dominant crop grown in Kazakhstan and thus was used for the calculation of crop residues [31]. At harvest, around 90% of wheat biomass is aboveground [64]. The energy contained in crop residues is generally less than that of pasture, and a value of 6 MJ/kgDM (using wheat as the base reference) was used for croplands [63, 65]. The total energy available for grazing from croplands was calculated to be 293 PJ. The grassland map and cropland map were then merged to produce a map of grazing supply (Fig 2).

### Grazing demand

**Fig 3:**
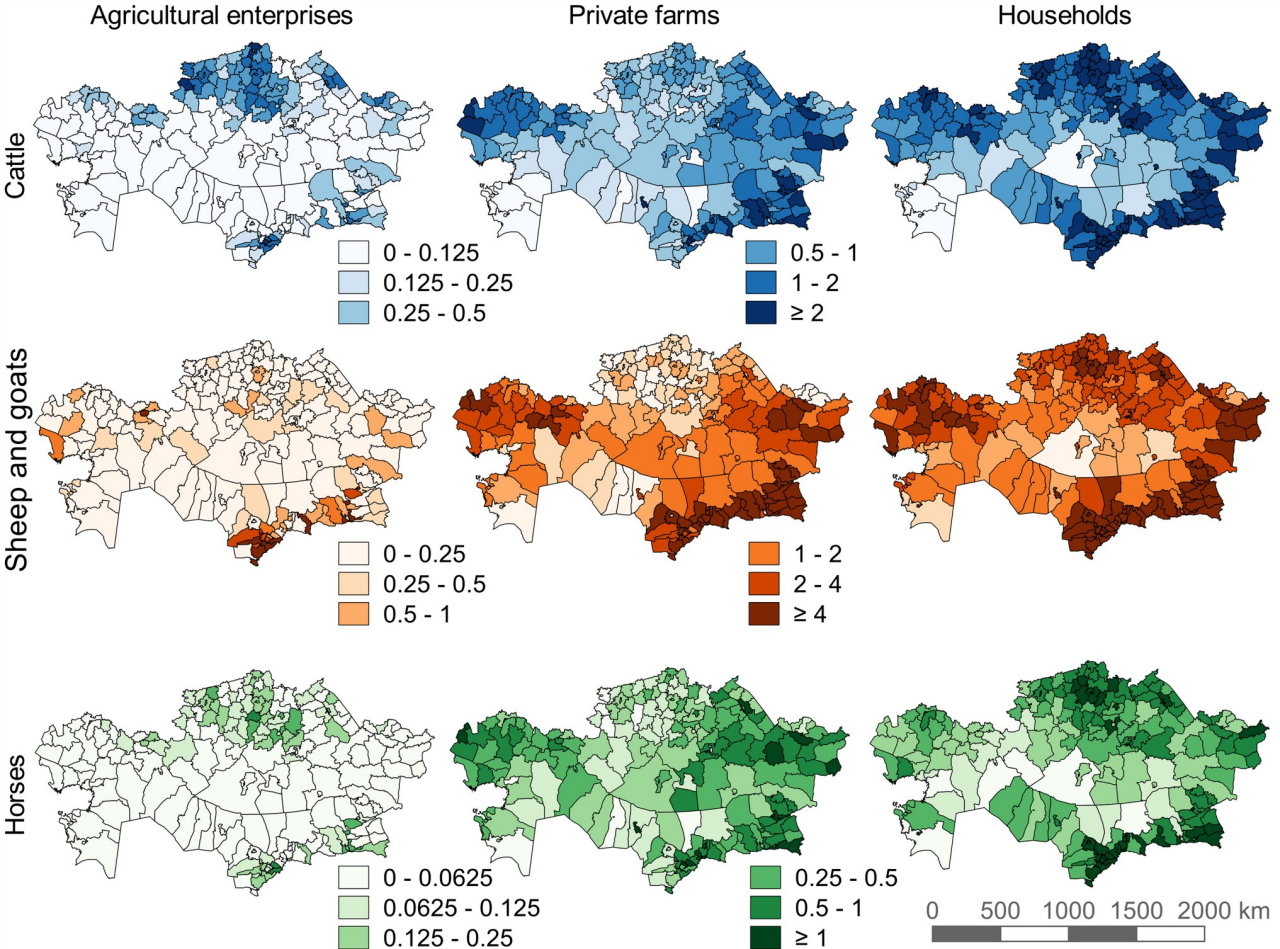
Livestock density (head/km^2^) at the district level for the three farm types in Kazakhstan for the year 2015 [31]. Livestock in Kazakhstan are not distributed evenly across space, nor across the three farm types.

To calculate grazing demand, we gathered data on livestock numbers, fodder yield and fodder consumption at the district level (2^nd^ level administrative division) for 2015 from the Kazakh National Statistics Agency (Fig 3) [31]. In Kazakhstan, there are 200 district-level units, consisting of districts (*rayons*) and city administrative units (*gorodskie administratsii*). Livestock nutritive requirements were taken from recommended values published in a livestock nutrition handbook by KazAgroInnovation [66], a subordinate of the Kazakh Ministry of Agriculture. These values are specific to Kazakh livestock at different stages of growth and for different animal functions (e.g., beef heifers for breeding vs. beef heifers for finishing), as well as for different desired growth rates. Because sheep far outnumber goats in Kazakhstan, and because of their similar grazing characteristics and energy requirements, all goats were treated as sheep.

To calculate the total nutritive demand (Fig 1), we used information on the age group and animal function of the Kazakh livestock herds. The proportions of the different age groups and animal functions were available at the province (oblast) level for the different livestock types from the 2006 agricultural census [67]. As no newer or more detailed data exist, these proportions were applied to the 2015 numbers in our disaggregation equation (S2 Equation). Animal productivity differs depending on living conditions, and living conditions in Kazakhstan can broadly be defined based on the farm type. The differences in animal productivity, and thus energy demand, on the different farm types was estimated using different animal growth rates (g/day) as indicated in the handbook (S2 Table) [66]. A description of how the handbook values were used to calculate the different animal age and function groups can be found in S3 Table.

The fraction of total energy demand that is not met by fodder and therefore must be met by grazing (i.e., grazing demand divided by total demand) is called the grazing gap, and was obtained by subtracting the amount of energy that is consumed as fodder from the total demand [68]. The estimation of fodder consumption was not straightforward, as such statistics are reported consistently only for agricultural enterprises. Instead, gross yield of harvested fodder crops at the district level was used [31], and fodder consumption statistics were used in an equation (S3 Equation) to allocate fodder to the different livestock types using their relative proportions (i.e., proportion of total fodder consumption allocated to cattle, sheep, goats, pigs, poultry, horses, and camels). This was the only possible way to estimate the total amount of fodder consumed by grazing livestock in Kazakhstan. Fodder crop yields were converted to energy values using the Soviet system of “fodder units” [42], due to its easy conversion to MJ and its widespread use in Kazakh agricultural literature and statistical reporting (S4 Table shows the conversion rates used).

Table 1 details the inputs used to distribute the demand onto the supply as shown in Fig 1. When distributing the supply, it is important to acknowledge that only a fraction of the total NPP can be consumed by livestock. The Eisfelder et al. [47] map is a measurement of total NPP, which includes the portion that is belowground and unavailable to the livestock. We used the work of Propastin et al. [69], who found aboveground NPP in central Kazakhstan to be on average 77% of total NPP. In addition, a considerable portion of the aboveground NPP must be left to allow regrowth. Published values of recommended stocking rates and pasture utilization in similar climatic conditions suggest a maximum off-take rate of 40% of aboveground NPP [70–72], and thus the maximum sustainable off-take rate was estimated at 40% of 77%, or 30% of total NPP.

**Table 1:** Summary of parameters used.

Input parameter (units in parentheses)	Spatial resolution	Reference period	Source
Livestock numbers	District	2015	KazStat [31]
Fodder production (Joules)	District	2015	KazStat [31]
Fodder consumption (Joules)	District/Province	2015	KazStat [31]
Nutritive requirements (Joules)		2008	KazAgroInnovation [66]
Herd age structure	Province	2006	KazStat [67]
Human population	Settlement	2009	KazStat [49]
Settlement location	Settlement	2016	Geofabrik [46]
District and municipal areas	District	2015	GADM [73], GIS-Lab [74]
Net primary production (gC/m^2^)	1 km	2003–2011	Eisfelder et al. [47]
Land cover	250 m	2009	Klein et al. [11]

Net primary production was taken as an average of the annual products from 2003 to 2011.

Obviously, not all pasture is grazed at the maximum sustainable off-take rate. Mapping actual grazing intensity requires estimating the variation in off-take rate on a spatial scale. To map variation in off-take rate using our model, we first ran the model under a range of eleven different off-take rate assumptions (5% increments from 10%-60%). We then calculated the maximum distance from each settlement that each livestock type needed to fulfill their grazing demand under each off-take rate. To determine an accurate off-take rate for each settlement, we used the maximum grazing distances for cattle in households as the defining variable, as they, along with sheep and goats (which are distributed before cattle), are the most restricted by distance from settlement. We chose 10 km as the maximum distance for cattle in households based on the findings of Kamp et al. [50]. We grouped settlements into their districts, and for each district, we selected the lowest off-take rate that corresponded to the median of maximum grazing distances for cattle in households being less than 10 km. In cases where districts had no reported cattle in households, sheep and goats in households instead were used (districts without either of these had no grazing livestock of any kind). The model was then run again, with settlements maintaining their determined off-take rate.

### Production potentials of meat and milk

We estimated the potential to increase production of meat and milk in Kazakhstan based on the efficiency with which pasture is being used. To estimate pasture use efficiency, we took meat and milk production in 2015 from the national statistics [31]. We calculated pasture requirement from the model results and estimated the yield of meat and milk (tons per km^2^ utilized) for the different livestock types. Our model results do not differentiate between beef and dairy cattle, so we made an adjustment based on the relative proportions of beef and dairy cattle. The fraction of cattle classified as dairy in 2015 for agricultural enterprises, private farms, and households was 0.40, 0.50, and 0.85, respectively [31]. We multiplied the land requirement by this fraction as a rough estimate for the area used by dairy cattle. We then divided milk production by the adjusted land requirement to estimate milk productivity.

We used the calculated land use efficiencies to estimate production potential. First, we made a conservative assumption that all land within 10 km of a settlement could currently be utilized. Therefore, unutilized land within 10 km of a settlement was considered for potential expansion. The modeled off-take rates were used to calculate the number of additional livestock that could be supported. Second, we proposed a scenario where pasture was grazed at its maximum sustainable intensity (30% off-take rate), and calculated the resulting unutilized area within 10 km of a settlement. Using a less conservative assumption that all land within 20 km of a settlement could be utilized, we repeated the previous two calculations. Potential increase in beef production was calculated with the assumption that all additional livestock were beef cattle. Similarly, for potential increase in milk production, we assumed that all additional livestock were dairy cattle. Therefore, the results presented are “either-or”, and the reality likely falls somewhere in between.

## Results

### Grazing gap and demand distribution

**Fig 4:**
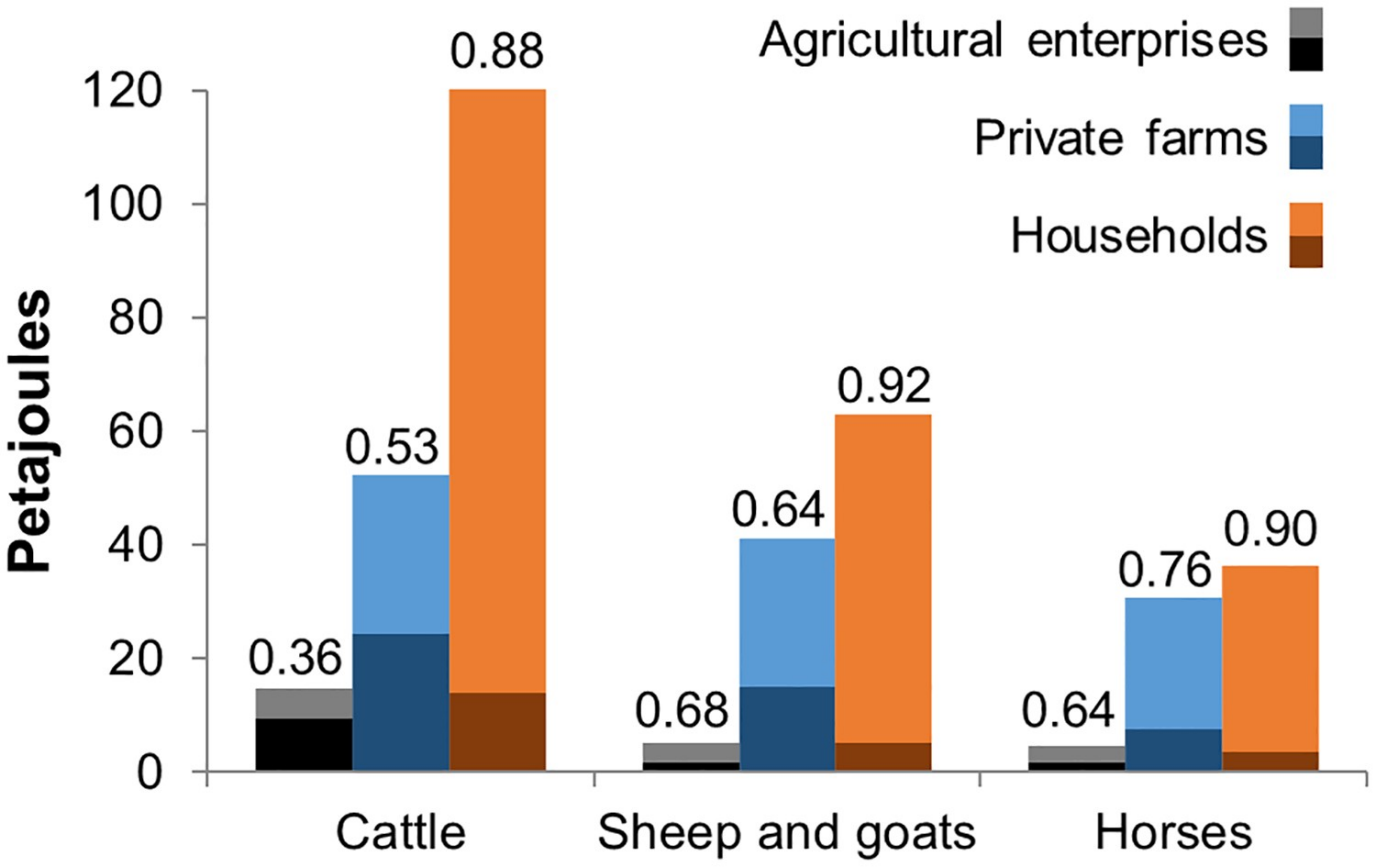
Energy balance for Kazakh grazing livestock in 2015. The darker bottom portion of each bar is the fodder supply, and the lighter top portion is the remaining demand that must be acquired from grazing. The total demand is represented by the full bar height. Fractions above each bar show the grazing gap (grazing demand divided by total demand). Nutritive demand information is from KazAgroInnovation [66] and supply statistics from KazStat [31].

The grazing demand was calculated for each animal and farm type combination (Fig 4). The total energy demand by all livestock types in 2015 (the sum of all bars in Fig 4) was 368 Petajoules (PJ). The grazing gap is displayed above each bar as the fraction of total energy demand obtained through grazing. Of the three livestock types, the grazing gap is lowest for cattle, and of the three farm types, the grazing gap is lowest for agricultural enterprises, with the lowest being cattle on agricultural enterprises. This is due to cattle on agricultural enterprises receiving more and higher-quality fodder than other livestock and on other farm types. The total amount of energy supplied by fodder was 82 PJ (sum of all darker portions of the bars), leaving 286 PJ to be obtained through grazing.

**Fig 5:**
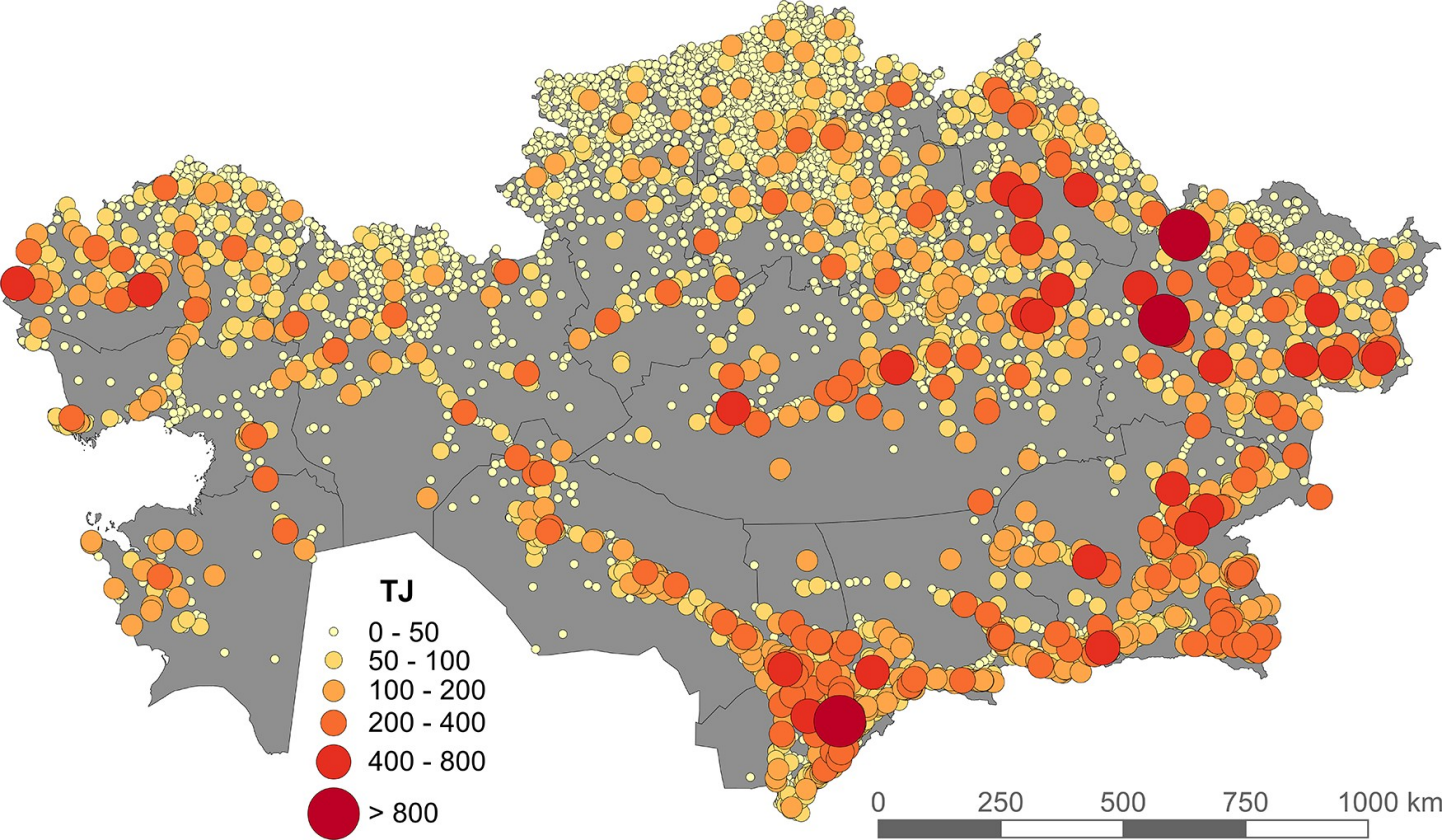
Grazing demand (in Terajoules, TJ) in 2015 disaggregated to settlements (all farm types combined).

Fig 5 shows the total grazing demand of all livestock types for each settlement in Kazakhstan. Grazing demand is not distributed evenly across the country. In the north, the demand is large, but dispersed across many settlements, whereas in the south it is also large, but concentrated in relatively fewer settlements. The center and southwest have both few settlements and little grazing demand.

### Off-take rate

**Fig 6:**
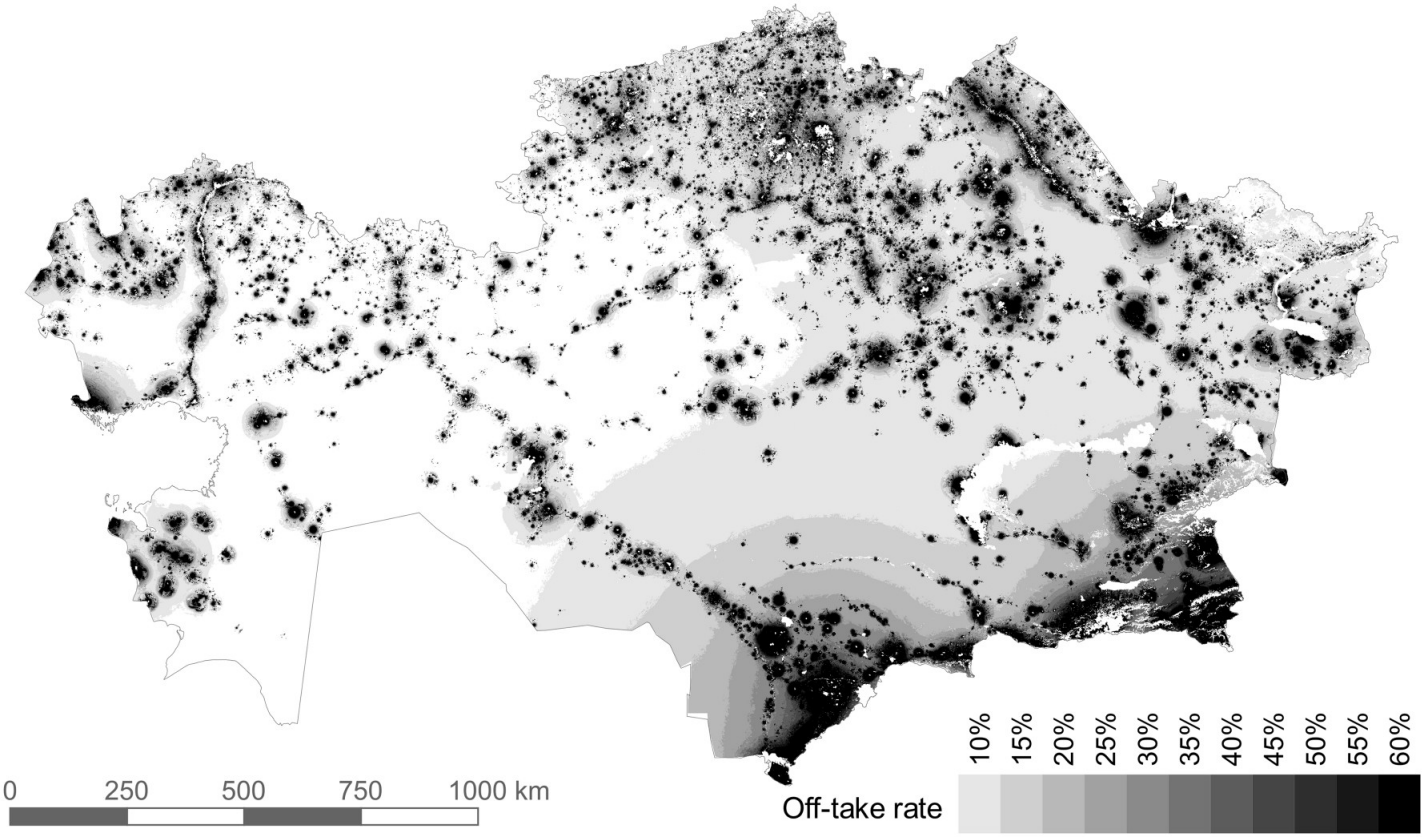
Grazing extent by all livestock under varying off-take rates. The image is a superimposition of the eleven model runs. The map of each individual off-take rate includes the area of all higher off-take rates.

**Fig 7:**
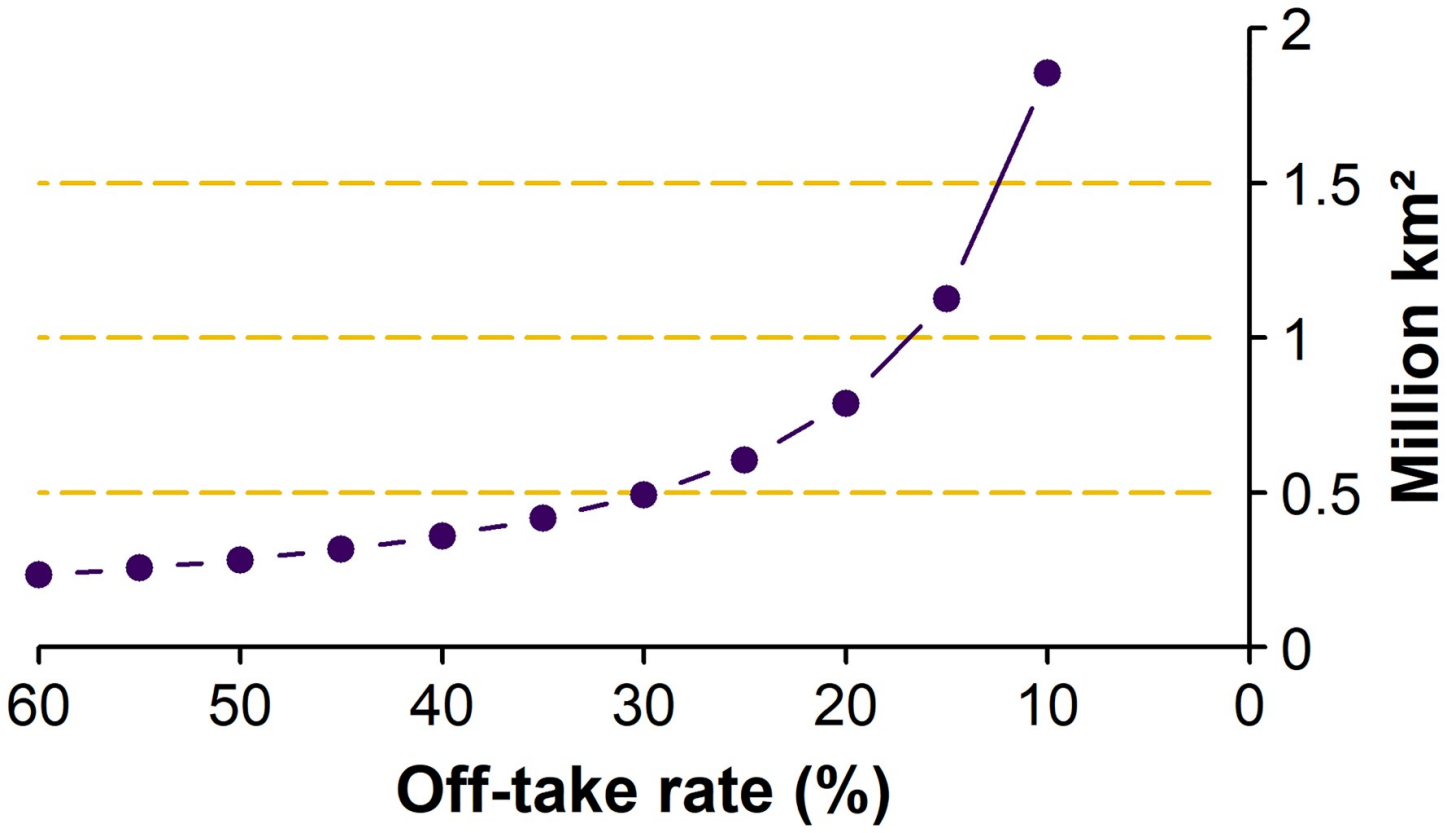
Area required by grazing livestock depending on the percent of biomass off-take.

Off-take rate is not uniform across Kazakhstan. The grazing demand for 2015 was 7.5% of the total biomass supply (when converted to energy)—i.e., if the off-take rate were 7.5%, all available land would be utilized. We tested the sensitivity to off-take rate by running the model with eleven different off-take rates, from 10% to 60% (5% increments) (Fig 6). As the off-take rate decreases, the area required for grazing increases exponentially (Fig 7). In our results, all off-take values used are as a percent of total available NPP.

### Grazing distances and pasture extent

**Fig 8:**
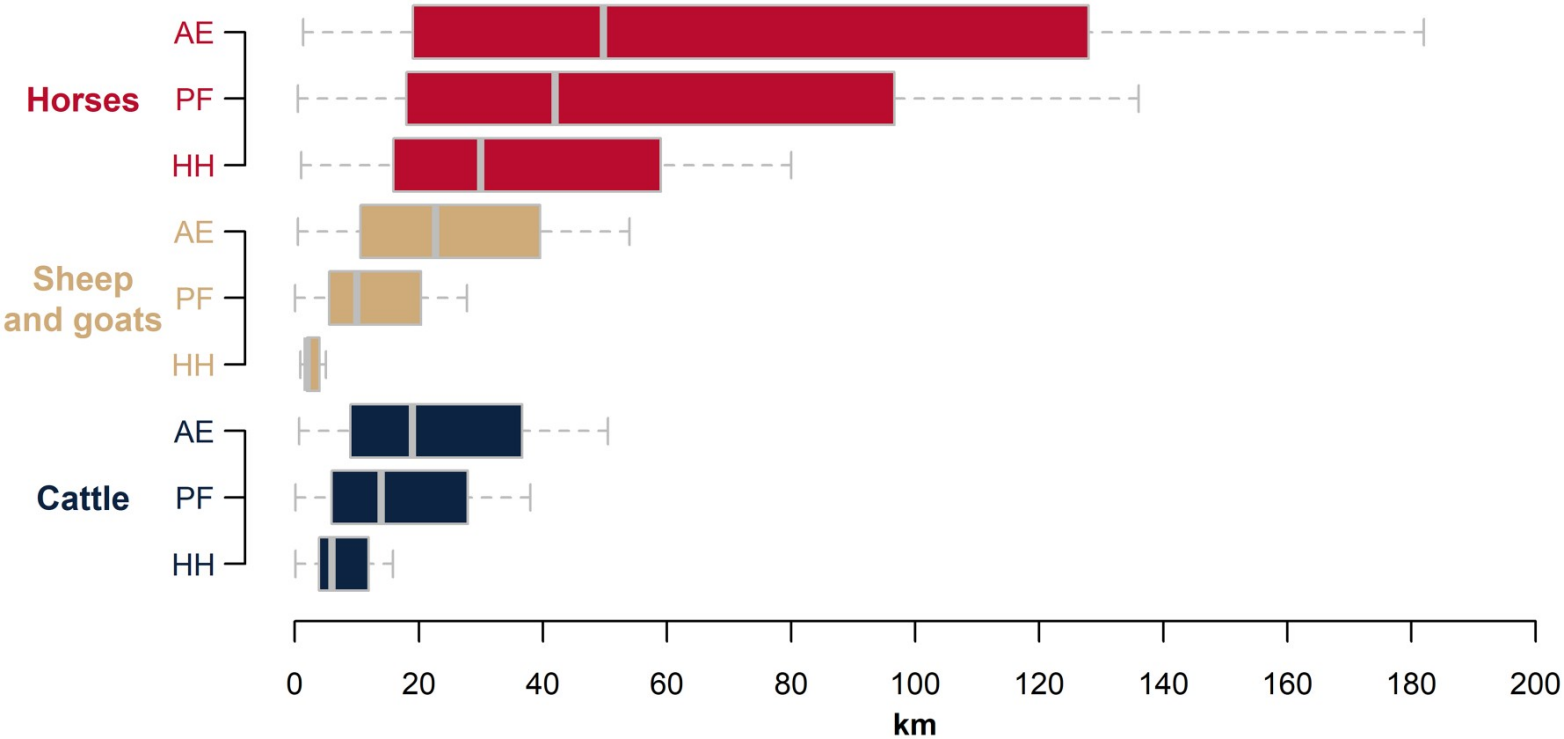
Maximum grazing distances of the variable off-take rate map. Box shows 50% of settlements (with median), whiskers are ½ inter-quartile range. AE: agricultural enterprises, PF: private farms, HH: households.

The maximum distances traveled by household cattle under each off-take rate assumption were analyzed at the district level to determine the average off-take rate in each district. The model was re-run with variable off-take rates to derive the maximum grazing distances. Fig 8 shows the median and quartiles of these distances by animal and farm type. A smaller quartile range on the left-hand side of the median for every livestock type is a result of the distances being skewed by relatively few settlements with a large livestock population located close to one another, most notably in southcentral Kazakhstan (Fig 5). Most settlements had much shorter maximum grazing distances, within 6 km for cattle, sheep, and goats in households, and within 15 km for cattle, sheep, and goats on private farms. Despite being distributed later, cattle on agricultural enterprises were found to have lower maximum grazing distances than sheep and goats on agricultural enterprises. This was due to agricultural enterprises specializing in cattle production being located mainly in the north in small settlements, whereas agricultural enterprises with sheep and goats were located mainly in the south and southeast in or near large settlements.

**Fig 9:**
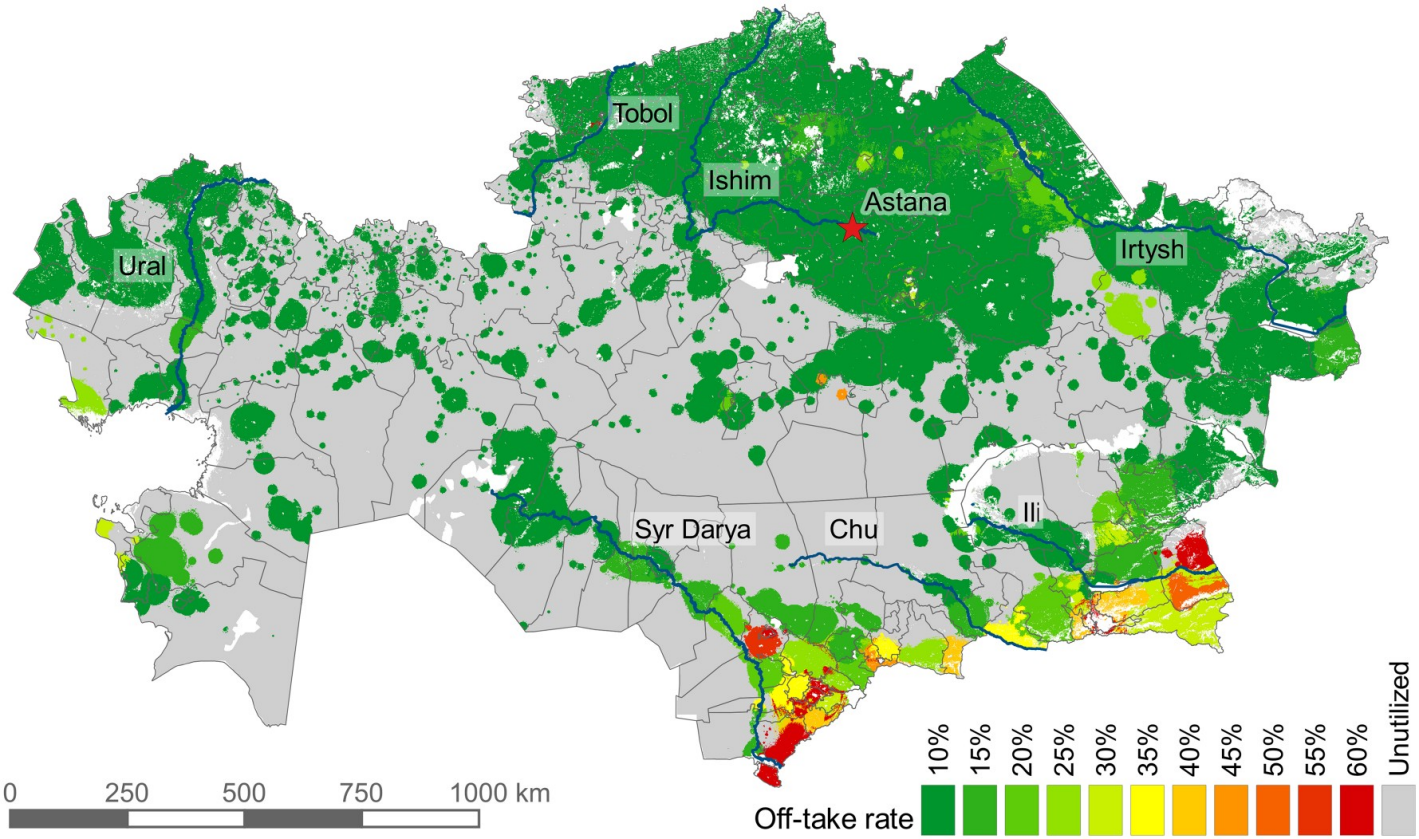
Distribution of grazing intensity in Kazakhstan for the year 2015. Off-take rate is the percent of total available biomass that is consumed. I.e. on croplands, it is the percentage consumed of the biomass that remained after harvest. Major rivers are shown in blue with names.

Fig 9 shows the land-use footprint of grazing livestock in 2015, using variable off-take rates at the district level. The area required was 1.22 million km^2^, 48% of the area theoretically available for grazing. While the off-take rate was determined at the district level, the result shows that off-take rates did not strictly adhere to district boundaries, as individual settlements are not obliged to graze within district boundaries. In the north, a relatively higher number of livestock are kept in private farms and agricultural enterprises, which are not as restricted as household livestock to the immediate vicinity of settlements. Thus, they can utilize distant pastures at lower off-take rates, and almost all of the north and northeast was utilized to some extent. The south and southeast showed less land being utilized, however at a much higher off-take rate. In the east, high NPP allows for lower off-take rates, and high numbers of private farm livestock can search out distant pastures. Two riparian pasture regions are clearly visible due to their course running through otherwise arid and semi-arid regions: the Ural in the far west and the Syr Darya flowing northwest out of the southern tip. The Chu River (to the east of the Syr Darya) is a historically important river that used to flow into the Syr Darya, but for many years has been diverted for irrigation and now disappears before reaching the Syr Darya. The Ili River in the southeast flows from the mountains of Tian Shan into Lake Balkhash, where it forms a large delta, providing grazing opportunities in an otherwise arid landscape.

### Production potentials of meat and milk

The summarization of utilized pasture made it possible to estimate the associated productivity of livestock production with regard to pasture use. Table 2 shows the area of pasture utilized and the respective productivity of meat and milk (production per km^2^ utilized). Meat productivity is highest for cattle on agricultural enterprises, but not by a lot. With the much smaller grazing gap for cattle on agricultural enterprises (Fig 4), one would expect the meat productivity (which doesn’t account for fodder) to be much higher. This is not the case because most cattle on agricultural enterprises are in northcentral Kazakhstan (Fig 3), where the off-take is low (Fig 9) and a lot of grazing on cropland (Fig 2) occurs. Both factors increase the land utilized by cattle on agricultural enterprises compared to other livestock and on other farm types, and thus decrease the relative meat productivity. Total beef production in 2015 was 417 thousand tons (kt), and total dairy milk production was 5.1 million tons (Mt).

**Table 2:** Pasture use, production, and productivity of meat and milk in 2015.

*Livestock type*	Farm type	Pasture utilized (mil. km^2^)	Meat production (kt)	Meat productivity (t/km^2^)	Milk production (kt)	Milk productivity (t/km^2^)
*Cattle*	AE	0.30	28.44	96.09	263.01	2213.05*
	PF	1.47	77.80	52.90	777.55	1048.46*
	HH	4.44	310.57	69.98	4101.06	1091.96*
*Sheep*	AE	0.10	3.16	32.52	0.04	0.38
*and goats*	PF	1.14	38.74	34.11	0.33	0.29
	HH	1.82	123.19	67.60	1.25	0.68
*Horses*	AE	0.17	2.17	12.94	0.69	4.14
	PF	1.35	25.01	18.47	9.76	7.21
	HH	1.44	74.26	51.44	15.42	10.68

Meat and milk production statistics from KazStat [31]. These numbers do not account for land used for fodder production.

*Adjusted for relative proportion of cattle in dairy production. AE: agricultural enterprises, PF: private farms, HH: households.

For sheep, goats, and horses, meat productivity is highest in households. For sheep and goats, this is due to sheep on agricultural enterprises and private farms primarily being raised for wool, with meat only a byproduct. Similarly, for horses, most meat production is done at the household level. Regarding milk production, cattle on agricultural enterprises are clearly the most land productive (when adjusted for the proportion of dairy production). Milk productivity is very low for sheep and goats, with almost all production coming from the very few goats in the country. Horse milk productivity is somewhat higher, because of the demand for the traditional horse-milk drink *kumys*, which is produced mainly at the household level.

**Fig 10:**
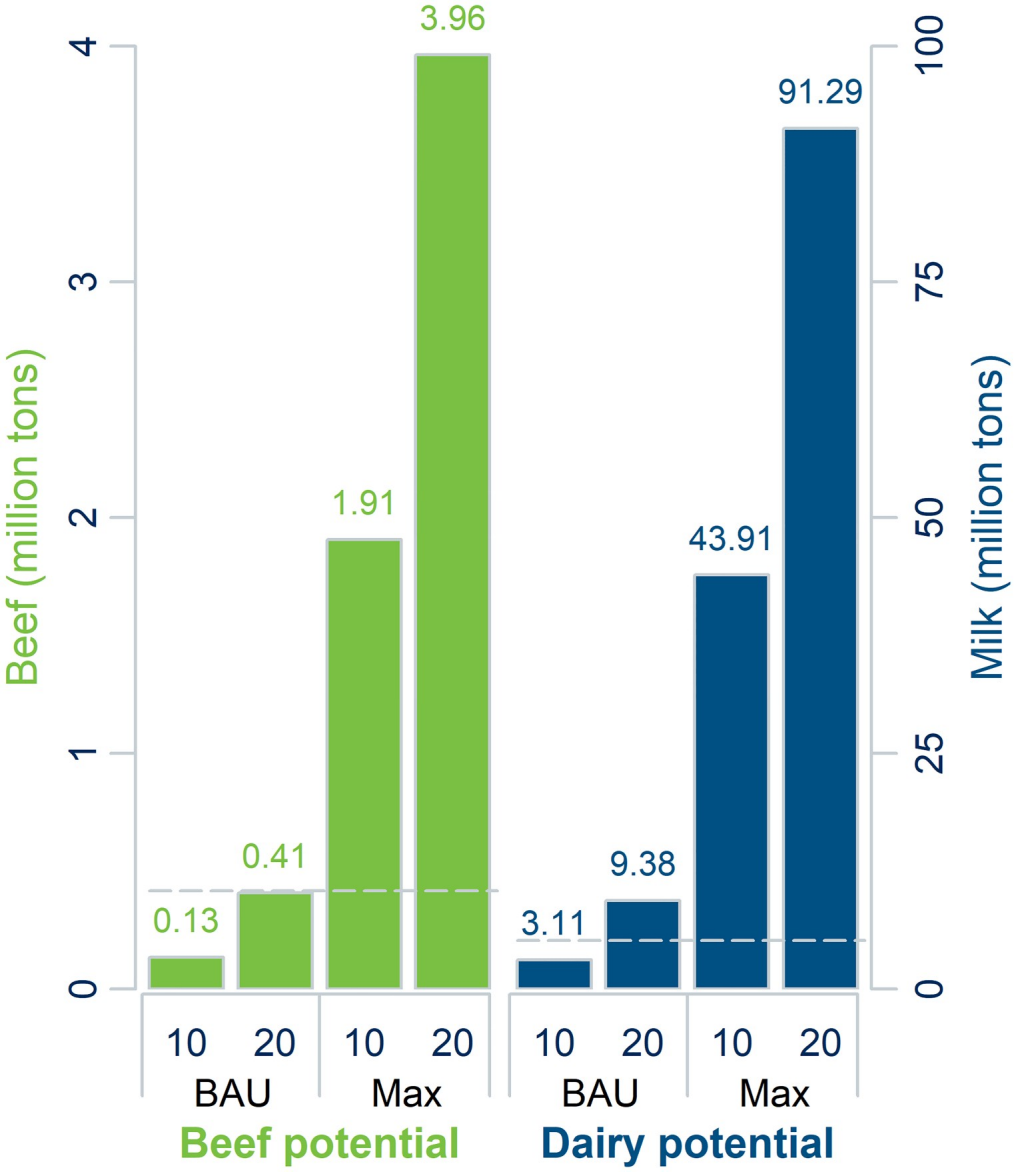
Production expansion scenarios. Additional beef and milk production under scenarios of business-as-usual (BAU, based on 2015 levels) and the maximum sustainable (30%) off-take rate, considering unutilized lands within 10 km of a settlement as well as within 20 km of a settlement. Based on 2015 productivity levels of agricultural enterprises (Table 2). The dotted line shows beef and dairy production in 2015.

We produced a conservative estimate for increased production potential by implementing the scenario where all land within 10 km of a settlement is utilized. Assuming the estimated off-take rates shown in Fig 9 as business-as-usual (BAU), the additional pasture utilized was 0.14 million km^2^, with an associated energy of 29.9 PJ. Assuming a proportional increase in fodder production (i.e., that the grazing gap remains the same), if the additional 0.14 million km^2^ of pasture was used entirely for cattle on agricultural enterprises, beef production could be increased by 0.13 Mt, an increase of 31% (Fig 10). Conversely, if all expansion was used for dairy on agricultural enterprises, dairy milk production could be increased by 3.11 Mt (above 2015 level) under the business-as-usual scenario, an increase of 60%. These are conservative estimates, as off-take rates were very low for most settlements (Fig 9). If all land within 10 km was used at its maximum sustainable off-take rate (30%), and if all additional livestock on pasture within 10 km were cattle on agricultural enterprises, beef production could be increased 1.91 Mt (above 2015 level), an increase of 457%. By comparison, Brazilian beef exports in 2013 totaled 1.25 Mt [75]. Hence, Kazakhstan has the potential to become one of the leading beef exporters in the world. If the radius of land around a settlement that can be utilized was increased to 20 km, with business-as-usual off-take rates, and if all additional pasture was utilized by cattle on agricultural enterprises, beef production could be increased by 0.41 Mt (98%). Assuming maximum sustainable off-take rates within 20 km, this estimate increases to 3.96 Mt.

## Discussion

This model is a new and unique way to analyze pasture use. As such, it cannot be compared directly to existing products. Most maps of livestock distribution or pasture extent are at the global scale, and we endeavored to create a more accurate map by focusing on the regional scale. We used detailed information on livestock nutritive requirements to calculate the total energy demand and we used district-level data on fodder supply to determine the grazing gap and grazing demand. To measure the energy available for grazing, we obtained NPP estimates as opposed to using suitability indices, which allowed us to estimate grazing intensity instead of probability of pasture occurrence or stocking density. Based on extensive literature review and field experience, we defined regional grazing practices specific to different farm and animal types to better distribute grazing demand. The result is a model that can distribute pasture utilization at fine spatial scales.

### Comparison to global products

We compared our results visually to global products. The Gridded Livestock of the World (GLW) [22] shows very dense populations of cattle, sheep, and goats in southcentral and southeastern Kazakhstan, whereas the north and northeast is less dense, but fairly uniform across a larger area. This agrees with the high off-take rates calculated in the south and the low off-take rates in the north, which results in very large continuous pasture in the north (Fig 9). More recently, Nicolas et al. [76] improved on the GLW by, among other things, preventing the GLW from placing livestock where humans are absent, which was also the aim of our model. However, they found no systematic improvements, likely due in part to their coarse distinction between urban and rural areas. The definition of urban and rural areas (and the presence of livestock in each) varies greatly between countries, and using one gradient for the entire world is unlikely to produce accurate results.

Compared visually to Erb et al. [14], our model found less utilized pasture. This was due to the subtractive method used by Erb et al. [14]—if land was not used for crops or anything else and as long as it was above the precipitation threshold, then it was considered grazing land. That qualifies almost all land in Kazakhstan as grazing land, including lands—such as in central Kazakhstan—where few, if any, livestock exist. However, Erb et al. [14] also published a grazing suitability map, which shows most of Kazakhstan, save for the far south and southeast, to be in the lowest suitability class. Though suitability is not necessarily related to utilization, the areas of high suitability (the south and southeast) match our areas of high off-take rates. Overall, the four global grazing land-use maps reviewed in Erb et al. [12] have low agreement in Kazakhstan, and especially in the drier central and southwest part of the country, where the difference in estimates of grazing area range by up to 100%. In another study based on Haberl et al.’s [77] estimation of Human Appropriation of NPP, Erb et al. [12] estimated the grazing intensity as a percent of actual NPP, and almost all of Kazakhstan is in the 0–10% range. Only in the southeast and especially in the southcentral does grazing intensity increase, up to about 50%, which is in line with Fig 9.

Fetzel et al. [68] combined the efforts of the previously described global pasture extents [14–17] with the GLW [22], grazing suitability [14], Human Appropriation of NPP [77], Moderate-resolution imaging spectroradiometer net primary productivity [78], and feed intake estimates [79–81] to create a global, gridded map of grazing intensity. Shortcomings in the grazing intensity distribution include the use of FAO statistics for livestock numbers, the resolution of the 2007 GLW (5 km), as well as the spatial scale of feed intake estimates (usually national level) [68]. These are general issues with global-scale models, which underline the usefulness of regional-scale approaches, as long as these can make use of data with higher spatial and temporal resolution. Further comparison to our results can be found in S3 Fig. As regional studies of the spatial distribution of grazing intensity do not exist, we compared our results to the global product by Fetzel et al. [68], cut to the area of Kazakhstan (S3a Fig). While the resolution of the maps is not comparable, the suitability algorithm employed by Fetzel et al. [68] resulted in a similar relative distribution of grazing intensity. The takeaway from this comparison is that our maps agree in the areas of high, and low, grazing intensity. There are a number of reasons why the results are not directly comparable, not the least of which being that Fetzel et al. measured grazing intensity in 2000, when grazing livestock numbers were 57% of their 2015 levels [31]. Also, the assumptions and inputs used in our model were specific to Kazakhstan. Our calculation of grazing gap (Fig 4) produced a much higher grazing demand in Kazakhstan than the average grazing demand calculated in Herrero et al. [81] for the region defined as the Commonwealth of Independent States. Additionally, our search parameters—using settlements as “home bases”—resulted in a smaller, more intensely grazed area. One noticeable inconsistency between the two maps is in the east. This is likely due to the very high NPP in the region, which would make it seemingly ideal for intense grazing. However, there are relatively few livestock in the region (which is only seen in district-level statistics), and much of the far eastern parts are covered in forest, which were not available for grazing in our model.

### Validation

For this research, we combined remotely sensed NPP, livestock statistics, feed recommendations, and allocation rules. Each of these components may contribute a degree of uncertainty. For instance, official statistics can be subject to manipulation and inconsistent measurements, particularly during the transition period. We utilized 2015 livestock numbers, after the reformation of reporting livestock statistics that occurred in 2010–11, which included livestock tagging. Similarly, actual feed consumption may vary according to individual production costs, output, and available fodder resources or other endogenous factors, such as experience and background of the farmer. Yet, agricultural producers tend to follow the recommendations of the extension services and the Ministry of Agricultre.

McNaughton et al. [82] summarize the pitfalls associated with ground-based measurements of grazing intensity, mainly—but not limited to—the difficulty in measuring plant growth, removal, and re-growth over a season. More recently, remote sensing-based global pasture products acknowledge the inherent difficulty in detecting pasture existence, much less determining grazing intensity [83, 84]. Grazing, especially low-intensity grazing, can leave almost no detectable signature. Adding the mobility of livestock and differing land covers, detection and measurement of grazing intensity is extremely difficult. As elaborated by Verburg et al. [85], improving or re-thinking the current methods of ground-truthing is a very immediate need in land use and land cover products and a cause for future research. Our research combined satellite-derived data (NPP) with sub-national livestock statistics. This makes our approach a member of the group of methods described by Kuemmerle et al. [10] that, while not ground-truthed themselves, use ground-truthed products to disaggregate spatially-aggregated data, a group that also includes the work by Neumann et al. [86] and the GLW [21–23].

### Targeting areas for livestock expansion

Expanding on the GLW, Chang et al. [87] estimated the potential livestock density for Europe and found that virtually everywhere there is large room for expansion. However, competition for land use in Europe is very high, and expansion is unlikely to occur at a large scale. This is not the case in Kazakhstan, where land is abundant and much of it is not utilized or utilized at low intensity levels. Increases in livestock mobility through improved infrastructure can reduce localized overgrazing and allow pastoralists to regain access to the vast steppe that is currently out of reach. Areas high in NPP, but with low off-take rates and relatively low livestock numbers, can be found in the east and in the northwest of Kazakhstan. These regions could be the easiest targets for increases in livestock production. In the south, there appears to be less pasture utilization in terms of area, but that belies the much higher off-take rates. It is possible that a 10 km threshold for household livestock is too restrictive in the south, where a much higher proportion of livestock are kept on household farms, and some medium-range seasonal migration is known to still occur [88]. This would result in more utilized pasture, with lower off-takes rates. In the north, a much higher proportion of livestock are held on agricultural enterprises, and the relatively low numbers of household livestock contribute to the low calculated off-take rate, usually 10%. This forces the agricultural enterprise cattle to range at large distances, further than 100 km for several settlements in Akmola Province. While this is certainly possible for agricultural enterprises with labor and capital resources, it is more likely that they graze closer to the farm at higher off-take rates. It is well-documented that overgrazing of pasture is still a feature of Kazakh livestock production, especially around settlements [89]. This would result in an overall smaller utilized pasture in the north, with higher off-take rates.

### Notes on model inputs and assumptions

In the estimation of maximum sustainable off-take rate, we used the findings of Propastin et al. [69], based on 14 test sites in Karaganda Oblast. While they have corroborated their results with other similar studies, in fact the proportion of above- to belowground NPP varies depending on the plant species, soil type, soil texture, and climate [90]. To date, robust datasets that map the spatial distribution of above- and belowground NPP in Kazakhstan are lacking. In similar fashion, no countrywide spatial datasets exist on the energy content of predominant species. In our model, a distinction was made only between cropland and grassland, and another improvement on the energy supply side would be to distribute spatially energy content based on predominant plant species. Including the spatial heterogeneity of the above- and belowground NPP and the plant energy content would improve the accuracy of our model’s estimation of grazing supply.

Our study found an overall grazing gap of 78%. Erb et al. [12] report the overall grazing gap for 11 world regions, with Central Asia and the Russian Federation having an aggregated grazing gap of around 40%. However, Russia has a large amount of production on primarily mixed feedlot systems and thus a low grazing gap, and similar climatic regions to Kazakhstan, such as Australia and Sub-Saharan Africa, have grazing gaps between 70 and 80% [12]. We should note, in Kazakhstan, it is likely that there is substantial fodder production that is not being reported and is therefore unaccounted for in our model. Hay cutting by households and private farms on grassland is a common practice and usually goes unreported. At the same time, communication with local experts and farmers suggested that it is unlikely that fodder imports from other countries occur in significant quantities, which is supported in official statistics [25]. However, some limited amounts of high-energy fodder may be traded from districts with large cropland areas to districts with large grassland areas (and vice versa with hay moving from grassland-dominant districts to cropland-dominant districts), which has not been accounted for in our model. However, the degree of fodder transport between districts is unknown.

Also unknown is the extent to which livestock utilize crop residues. As residues may or may not be left in the field, there are no statistics on the yield of residues available for livestock. However, expert opinion and field experience suggested that there is wide-scale grazing of harvested lands, and a lack of physical boundaries (such as fences) led us to assume in our model that all crop residues were available to livestock. NPP from crop residues available to livestock was calculated to be 293 PJ. This is small in comparison with the grassland supply of 3537 PJ (less than 7% of the total energy supply, yet occupying 14% of the total area), but is a significant source of energy for livestock, particularly in northcentral Kazakhstan. Notably, the time of year that livestock forage on croplands changes from year to year. The wheat harvest can sometimes last into winter when weather conditions prevent earlier harvest. In our model, we do not account for this, making the implicit assumption that most residues from these late harvests will still be available for foraging the following spring, or for horses that graze year-round. Indeed, for an average annual grazing supply map such as we used, whether the residues are consumed in spring or in fall is moot.

The area utilized in 2015 covers much of the highest-productivity areas shown in Fig 2, and there are clear trends of pasturing near rivers and in other areas of high NPP (Fig 9). As the restriction to settlement location is much more important than the productivity of the pasture, this is a result of the location of the settlements and not the model search algorithm. Most settlements were founded because of their proximity to water or other biophysical characteristics that favor higher biomass productivity. Indeed, the average energy production on land within 10 km of a settlement in Kazakhstan is 1.75 MJ/m^2^, whereas land further than 10 km from a settlement averages 1.37 MJ/m^2^. This also supports the hypothesis of abandonment of pastures occurring mainly on marginal lands [24]. Additionally, analysis of Fig 7 shows that the off-take rate increases more than the expected function 1/x as off-take rate decreases, indicating lower productivity as the distance from settlement increases.

The model assumes that livestock are only located where people are located, similar to assumptions made by others [76]. While this is generally the case, livestock are not distributed evenly among the settlements, especially on agricultural enterprises. For example, agricultural enterprises hold a large number of animals and are commonly located in medium and small settlements, where they are the main source of jobs for the residents. Since some (but not all) small settlements have an agricultural enterprise, there is no way to place agricultural enterprises accurately within a district without preexistent knowledge of their location. This means that, in reality, herd sizes in small settlements are very unbalanced, whereas in the model, similarly sized settlements within a district receive equal numbers of livestock. Additionally, settlements that exist for a specific industrial purpose may have pasture distributed in the model where in fact no livestock are kept.

Briefly mentioned but unelaborated on is the role that access to water plays in livestock distribution. Wells have been an integral part of grazing migrations in Kazakhstan for centuries, and during Soviet times, thousands of new pump wells were installed to accommodate the regulated migrations of much larger herds [91]. Around these wells, outposts were erected and served as seasonal base camps for livestock herders. However, many of these outposts were abandoned after the collapse of the Soviet Union and the wells fell into disrepair [92]. There is currently no accessible countrywide data on the prevalence or location of working wells or occupied outposts. For this reason, wells and outposts were not considered in the distribution of livestock. Inclusion of these water sources as potential home bases for livestock would give the search algorithm access to grazing supply that was previously far from settlements, and thus it is possible that there is pasture utilized in remote areas not captured in the model.

### Livestock productivity on private farms

The low meat and milk productivity of cattle on private farms is surprising (Table 2), especially given that private farms have much lower grazing gaps than households (Fig 4). In the scenarios explored for Fig 10, a similar expansion on only private farms would result in an increase of 67 kt (16%), compared to 113 kt for agricultural enterprises. Through personal observation and anecdotal evidence, this lower productivity could be because private farmers do not have the resources necessary to provide proper nutrition to their livestock, resulting in suboptimal meat and milk production. Indeed, outside of agricultural enterprises, almost all fodder produced is hay, which is one of the least energy-rich fodder types. On agricultural enterprises, hay comprises 57% of fodder by mass, whereas on private farms hay comprises 95% of fodder by mass [31]. Additionally, in many regions private farms are essentially large households, and have similar grazing practices. In those situations, private farms at best share pasture with household livestock and at worst are pushed to the less productive lands far from the settlement. In the model, private farm livestock are always distributed after the same type of livestock in households, which biases private farm livestock to pastures with lower NPP, though across the country the effect should be rather small. Private farms are currently the most dynamic and fastest growing sector in livestock farming, but so far, their efficiency has not surpassed that of household farms.

## Conclusion

Here we have presented a new model to allocate livestock and pasture spatially, using a map of biomass production and information on animal types, farm types, and fodder intake. Our methods of delineating land used by livestock and estimating the intensity with which the livestock are using the land allow pinpointing the extent and location of underutilized grazing resources.

The model enabled a gridded estimate of the utilized pasture in Kazakhstan, which is a prime example of a country well suited to grazing livestock production. Kazakhstan’s dry continental climate also reduces the suitability of livestock production’s main competitor for land, crop production, making it a suitable target area for development of range-based livestock production. Our results show that despite relatively low natural productivity, ample capacity exists to increase livestock production in Kazakhstan because large areas are characterized by low pasture utilization and off-take rate, and available biomass resources could support many more grazing animals, especially in the east and the northwest (Fig 9). Under conservative estimates of grazing range constraints and with 2015 productivity levels, beef production could be increased by 0.13 Mt (31%) or milk production by 3.11 Mt (60%), or some combination. However, harnessing even a fraction of these potentials would necessitate infrastructure development measures, such as more, improved processing facilities and improved road networks and market access. Repaired wells and outposts would allow the rejuvenation of old migration patterns and would open up distant pastures for even more potential production increases.

This research is an important step forward in the field of livestock mapping. Our model uses much finer-scale inputs than other global-scale products, and a direct measurement of biomass production, enabling us to make a gridded estimate of pasture distribution based on the energy demand of the livestock. The search algorithm we created is easily transferable to other regions where livestock are restricted to a central point, but can be adapted to any region where grazing patterns can be defined. The result of our research can be used to find patterns in livestock distribution, and to target areas where the supply is underutilized. Moreover, our results help the spatial targeting of possible investments for expanding the production of grazing livestock, including assessing the tradeoffs of production expansion with greenhouse gas emissions and biodiversity conservation.

## Supporting information

S1 Equation The piecewise linear function used to estimate the number of livestock owners in a settlement based on the total settlement population. (DOCX)

S2 Equation The equation used to estimate the number of livestock in each age group in each district in 2015 using the more detailed 2006 agricultural census. (DOCX)

S3 Equation The equation used to estimate the amount of energy supplied by fodder to each livestock species in each district in 2015. (DOCX)

S1 Fig Piecewise function for the distribution of livestock to settlements based on human population. The steps are 95% from 0–1,000, 80% at 5,000, 30% at 10,000, 10% at 50,000, and 0.5% at 1 million residents (not shown), with linear interpolation. Values were determined from expert opinion and personal observation. Note that this does not affect the total number of livestock, only their distribution within a district.
(TIF)

S2 Fig A magnified view showing the result of the distribution model at the settlement level. Each unique color represents the land distributed to a unique settlement (black dots). This illustrates the operation of the search algorithm when settlements are in close proximity and their pasture requirements conflict.
(TIF)

S3 Fig Side-by-side comparison with the results of a) Fetzel et al. [68] and b) our model. Note that the units are not directly convertible. However, using very different methods and inputs, both maps show similar distributions of relative grazing activity. White areas in both maps represent unutilized area. a) Reproduced with permission of the authors.
(TIF)

S1 Table Assumptions made in the distribution model. (DOCX)

S2 Table Values used in the calculation of total energy demand [66]. The number of each livestock species are recorded at the district level for each farm type. Livestock numbers for each age group were recorded in the 2006 agricultural census at the regional level (in which cattle were further divided into beef and dairy) [67]. The number of livestock in each age group of each livestock species in each district was estimated by multiplying the number of each livestock species by the number in each species’ age group (in 2006) and dividing by the number of each livestock species (in 2006). Sheep and goat numbers were combined and sheep nutritive requirements were used, due to the low number of goats and their similar nutritive requirements.
(DOCX)

S3 Table Age groups used in the estimation of livestock energy demand. Restrictions are noted when the age group is a subset of the Age/Sex in S2 Table. Nomenclature of the age group is as follows (square brackets enclose string variables): [*Beef/Dairy*][Function]_[*age*]_[*reproduction stage*]. *–if applicable. Function–common name considering age, sex, and castration.
(DOCX)

S4 Table Conversion ratios used to convert kg to MJ [42]. Production of each fodder type is recorded at the district level for each farm type. Consumption of each fodder type by each livestock species is recorded at the regional level (all farm types combined). The consumption of each fodder type by each livestock species at the district level for each farm type was estimated by multiplying the production of each fodder type by the consumption of each fodder type by each livestock species and dividing by the total consumption of each fodder type by each livestock species.
(DOCX)
