# Supplementary material for: Modeling the spatial distribution of grazing intensity in Kazakhstan
Source: PLoS One. 2019 Jan 11;14(1):e0210051. doi: 10.1371/journal.pone.0210051 (PMC6329506; doi:10.1371/journal.pone.0210051)
Supplement: S1 Equation — (DOCX) [file pone.0210051.s001.docx]

$f\left( P \right)=\left\{ \begin{aligned} \begin{aligned} 0.95*P, &P\leq1000 \\ (0.95-\frac{0.15}{5000-1000}*\left( P-1000 \right))*P, &1000<P\leq5000 \end{aligned} \\ \begin{aligned} (0.8-\frac{0.5}{10000-5000}*\left( P-5000 \right))*P, &5000<P\leq10000 \\ (0.3-\frac{0.2}{50000-10000}*\left( P-10000 \right))*P, &10000<P\leq50000 \\ (0.1-\frac{0.095}{1000000-50000}*\left( P-50000 \right))*P, &50000<P\leq1000000 \\ 0.005*P, &P>1000000 \end{aligned} \end{aligned} \right.$(S1)

where:

$$P=settlement population (2009 census)$$

$$f\left( P \right)=settlement livestock owners$$
