# Supplementary material for: Modeling the spatial distribution of grazing intensity in Kazakhstan
Source: PLoS One. 2019 Jan 11;14(1):e0210051. doi: 10.1371/journal.pone.0210051 (PMC6329506; doi:10.1371/journal.pone.0210051)
Supplement: S2 Equation — (DOCX) [file pone.0210051.s002.docx]

$L_{mrla}=L_{mrl}*\frac{L_{oa}^{C}}{L_{o}^{C}}$ (S2)

where:

$$L=number of livestock$$

$$m=farm type$$

$$r=district$$

$$l=livestock type$$

$$a=age group$$

$$o=region where o\ni r$$

$$L^{C}=number of livestock in 2006 census$$
