# Supplementary material for: Modeling the spatial distribution of grazing intensity in Kazakhstan
Source: PLoS One. 2019 Jan 11;14(1):e0210051. doi: 10.1371/journal.pone.0210051 (PMC6329506; doi:10.1371/journal.pone.0210051)
Supplement: S3 Equation — (DOCX) [file pone.0210051.s003.docx]

$C_{mrfl}=P_{mrf}*\frac{C_{ofl}}{C_{of}}$ (S3)

where:

$$C=consumption \left( MJ \right)$$

$$P=production \left( MJ \right)$$

$$m=farm type$$

$$r=district$$

$$f=fodder type$$

$$l=livestock type$$

$$o=region where o\ni r$$
