# Supplementary figures and images for: Modeling the spatial distribution of grazing intensity in Kazakhstan
Source: PLoS One. 2019 Jan 11;14(1):e0210051. doi: 10.1371/journal.pone.0210051 (PMC6329506; doi:10.1371/journal.pone.0210051)

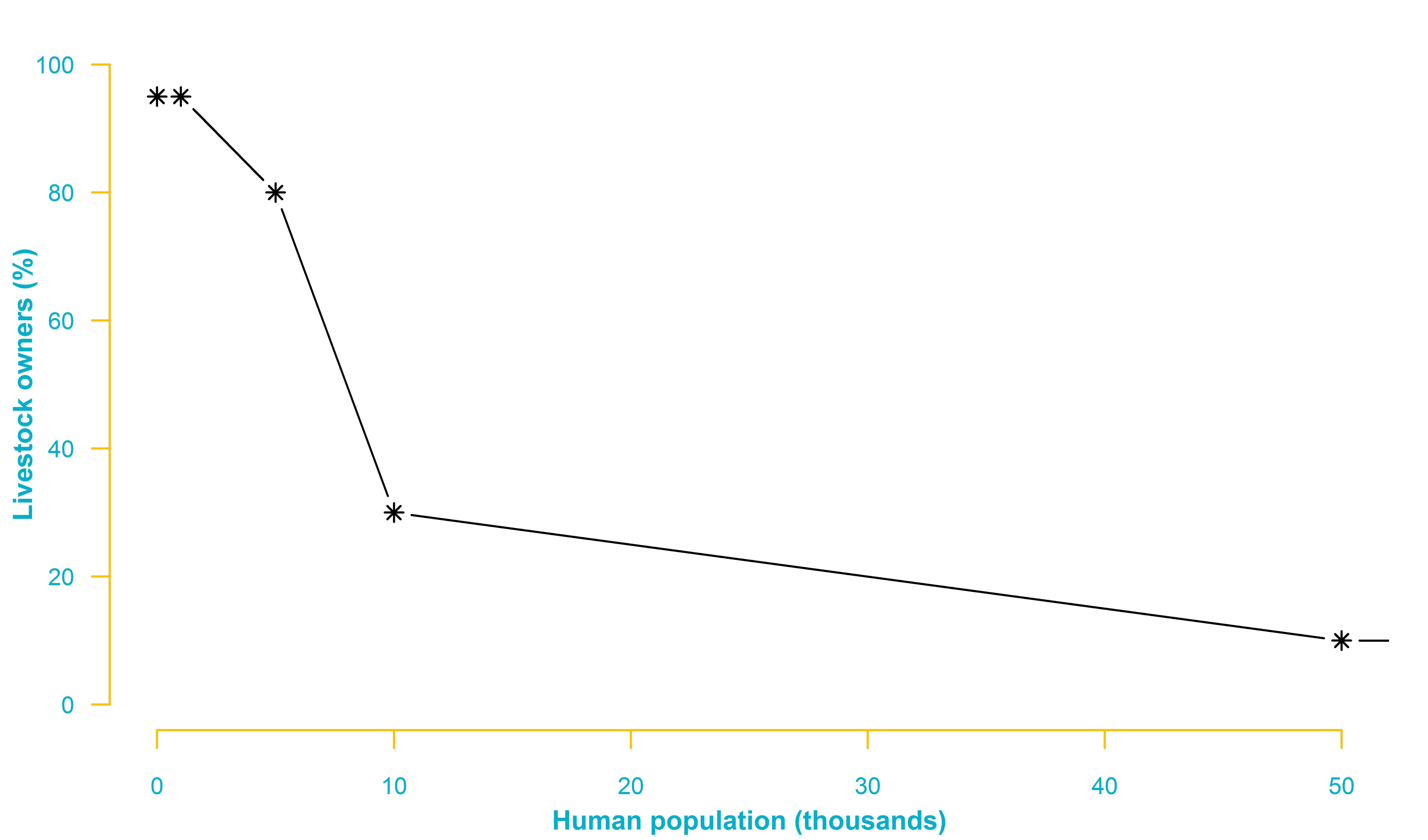

Supplement: S1 Fig — The steps are 95% from 0–1,000, 80% at 5,000, 30% at 10,000, 10% at 50,000, and 0.5% at 1 million residents (not shown), with linear interpolation. Values were determined from expert opinion and personal observation. Note that this does not affect the total number of livestock, only their distribution within a district. (TIF) [file pone.0210051.s004.tif]

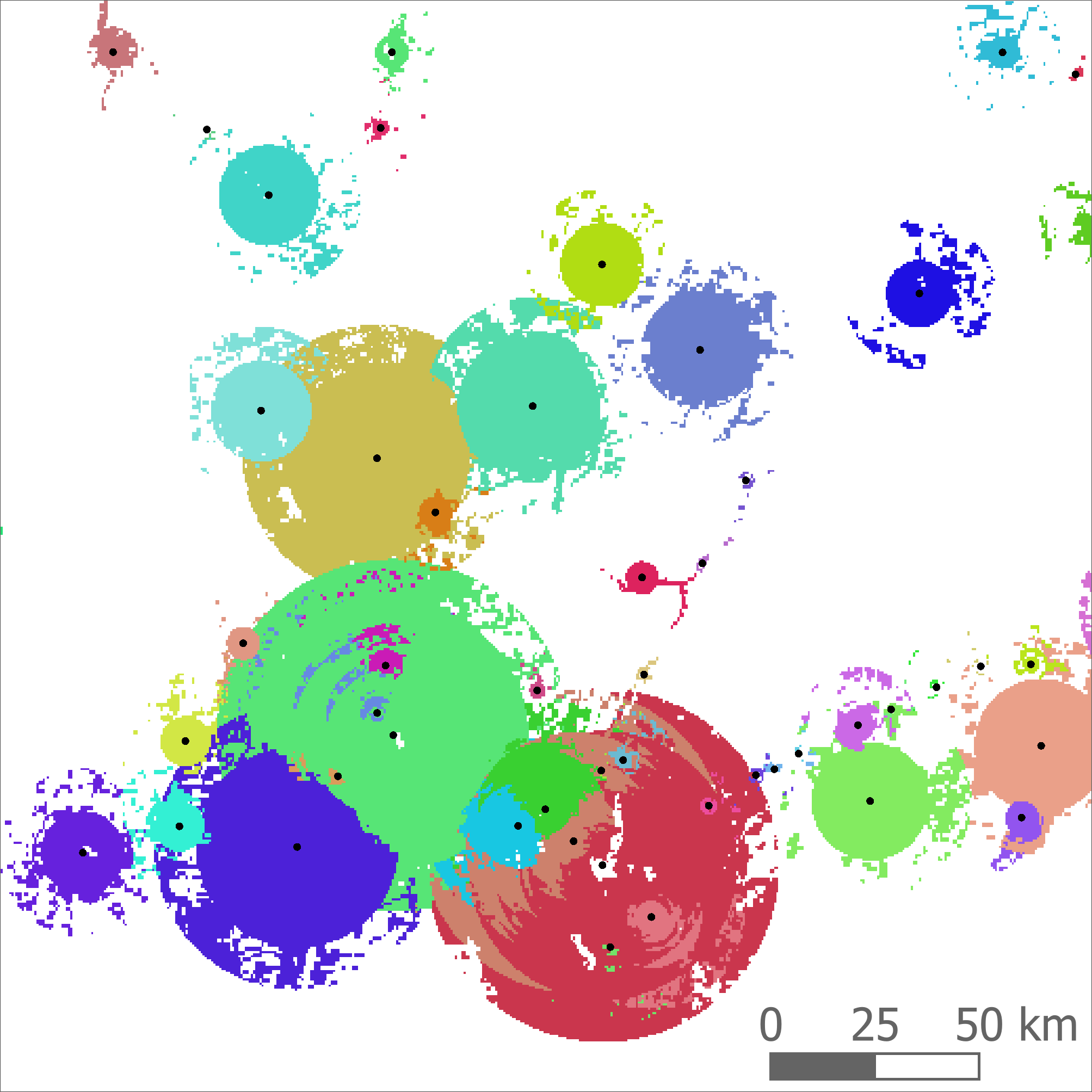

Supplement: S2 Fig — Each unique color represents the land distributed to a unique settlement (black dots). This illustrates the operation of the search algorithm when settlements are in close proximity and their pasture requirements conflict. (TIF) [file pone.0210051.s005.tif]

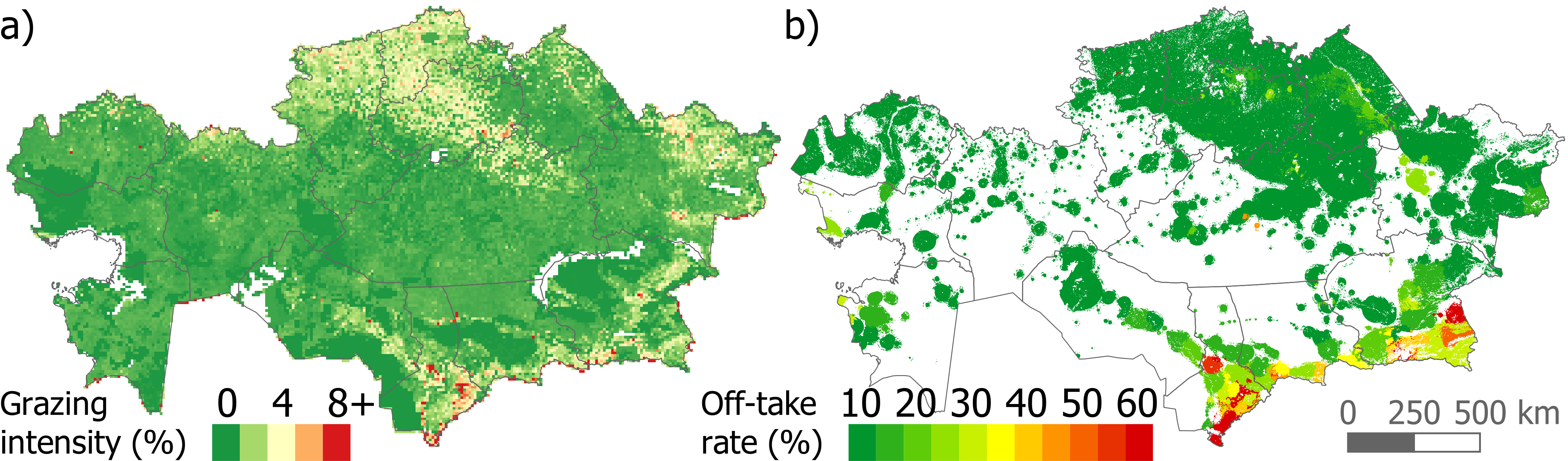

Supplement: S3 Fig — Note that the units are not directly convertible. However, using very different methods and inputs, both maps show similar distributions of relative grazing activity. White areas in both maps represent unutilized area. a) Reproduced with permission of the authors. (TIF) [file pone.0210051.s006.tif]
