# Supplementary material for: Modeling the spatial distribution of grazing intensity in Kazakhstan
Source: PLoS One. 2019 Jan 11;14(1):e0210051. doi: 10.1371/journal.pone.0210051 (PMC6329506; doi:10.1371/journal.pone.0210051)
Supplement: S1 Table — (DOCX) [file pone.0210051.s007.docx]

| Assumption | Source | Impact |
| --- | --- | --- |
| Livestock are located in settlements | [[38-40](#_ENREF_38), [43](#_ENREF_43), [50](#_ENREF_50)] | Nomadism is precluded, as are potential outposts and wells that are located far away from any settlement. Thus the areas furthest from settlements are the least likely to be distributed in this model |
| Livestock seek pastures with the highest productivity | [[45](#_ENREF_45), [91](#_ENREF_91)] | “Optimal forager” activity is severely truncated due to the introduction of search radii. Affects the distribution of individual livestock/farm types. The overall distribution is affected only at the edge of each settlement’s grazing area, where distributed area is clustered due to the priority of high-NPP values |
| Livestock graze on croplands | [[43](#_ENREF_43)] | The total energy available for grazing increased by 293 PJ, or 8.3%. Most of this energy was located in the northcentral, where cropland dominates. |
