# Supplementary material for: Modeling the spatial distribution of grazing intensity in Kazakhstan
Source: PLoS One. 2019 Jan 11;14(1):e0210051. doi: 10.1371/journal.pone.0210051 (PMC6329506; doi:10.1371/journal.pone.0210051)
Supplement: S2 Table — The number of each livestock species are recorded at the district level for each farm type. Livestock numbers for each age group were recorded in the 2006 agricultural census at the regional level (in which cattle were further divided into beef and dairy) [67]. The number of livestock in each age group of each livestock species in each district was estimated by multiplying the number of each livestock species by the number in each species’ age group (in 2006) and dividing by the number of each livestock species (in 2006). Sheep and goat numbers were combined and sheep nutritive requirements were used, due to the low number of goats and their similar nutritive requirements. (DOCX) [file pone.0210051.s008.docx]

| Type | Breed | Age/Sex | Period | Mass (kg) | Gain (g/d) | Milk (kg/d) | ME (MJ/d) |
| --- | --- | --- | --- | --- | --- | --- | --- |
| Cattle | Beef | Cows | 2 mos pre calving | 400 |  |  | 91 |
| Cattle | Beef | Cows | 2 mos pre calving | 450 |  |  | 98 |
| Cattle | Beef | Cows | 2 mos pre calving | 500 |  |  | 105 |
| Cattle | Beef | Cows | 2 mos pre calving | 550 |  |  | 112 |
| Cattle | Beef | Cows | 3-4 mos post calving | 400 |  |  | 113 |
| Cattle | Beef | Cows | 3-4 mos post calving | 450 |  |  | 117 |
| Cattle | Beef | Cows | 3-4 mos post calving | 500 |  |  | 122 |
| Cattle | Beef | Cows | 3-4 mos post calving | 550 |  |  | 127 |
| Cattle | Beef | Cows | 2nd half lactation | 400 |  |  | 92 |
| Cattle | Beef | Cows | 2nd half lactation | 450 |  |  | 101 |
| Cattle | Beef | Cows | 2nd half lactation | 500 |  |  | 109 |
| Cattle | Beef | Cows | 2nd half lactation | 550 |  |  | 117 |
| Cattle | Beef | Bulls | Not coupling | 800 |  |  | 83.2 |
| Cattle | Beef | Bulls | Avg load | 800 |  |  | 90 |
| Cattle | Beef | Bulls | Inc load | 800 |  |  | 110 |
| Cattle | Beef | Heifers | 9-10 mos | 244 | 575 |  | 69 |
| Cattle | Beef | Heifers | 9-10 mos | 260 | 675 |  | 78 |
| Cattle | Beef | Heifers | 11-12 mos | 277 | 575 |  | 76 |
| Cattle | Beef | Heifers | 11-12 mos | 300 | 675 |  | 85 |
| Cattle | Beef | Heifers | 13-14 mos | 311 | 575 |  | 83 |
| Cattle | Beef | Heifers | 13-14 mos | 340 | 675 |  | 94 |
| Cattle | Beef | Heifers | 15-16 mos | 345 | 575 |  | 90 |
| Cattle | Beef | Heifers | 15-16 mos | 380 | 675 |  | 104 |
| Cattle | Beef | Heifers | 17-20 mos | 413 | 575 |  | 105 |
| Cattle | Beef | Heifers | 17-20 mos | 420 | 675 |  | 112 |
| Cattle | Beef | Bull calves | 9-10 mos | 279 | 825 |  | 72 |
| Cattle | Beef | Bull calves | 9-10 mos | 285 | 925 |  | 79 |
| Cattle | Beef | Bull calves | 11-12 mos | 330 | 825 |  | 80 |
| Cattle | Beef | Bull calves | 11-12 mos | 340 | 925 |  | 88 |
| Cattle | Beef | Bull calves | 13-14 mos | 379 | 825 |  | 86 |
| Cattle | Beef | Bull calves | 13-14 mos | 396 | 925 |  | 94 |
| Cattle | Beef | Bull calves | 15-16 mos | 428 | 825 |  | 92 |
| Cattle | Beef | Bull calves | 15-16 mos | 451 | 925 |  | 102 |
| Cattle | Beef | Calves | 0-6 mos |  |  |  | 32 |
| Cattle | Beef | Calves | 6-9 mos |  |  |  | 54.6 |
| Cattle | Beef | Calves | 10-12 mos |  |  |  | 78.5 |
| Cattle | Beef | Calves | 13-15 mos |  |  |  | 93.5 |
| Cattle | Dairy | Cows | Milking 1st half | 400 |  | 6 | 72.5 |
| Cattle | Dairy | Cows | Milking 1st half | 400 |  | 10 | 106 |
| Cattle | Dairy | Cows | Milking 1st half | 400 |  | 14 | 127.5 |
| Cattle | Dairy | Cows | Milking 1st half | 400 |  | 18 | 149 |
| Cattle | Dairy | Cows | Milking 1st half | 400 |  | 22 | 172.5 |
| Cattle | Dairy | Cows | Milking 1st half | 400 |  | 26 | 197.5 |
| Cattle | Dairy | Cows | Milking 1st half | 400 |  | 30 | 227.5 |
| Cattle | Dairy | Cows | Milking 2nd half | 400 |  | 5 | 61 |
| Cattle | Dairy | Cows | Milking 2nd half | 400 |  | 7 | 83.5 |
| Cattle | Dairy | Cows | Milking 2nd half | 400 |  | 9 | 100.5 |
| Cattle | Dairy | Cows | Milking 1st half | 500 |  | 6 | 79.5 |
| Cattle | Dairy | Cows | Milking 1st half | 500 |  | 10 | 115 |
| Cattle | Dairy | Cows | Milking 1st half | 500 |  | 14 | 137 |
| Cattle | Dairy | Cows | Milking 1st half | 500 |  | 18 | 158 |
| Cattle | Dairy | Cows | Milking 1st half | 500 |  | 22 | 180.5 |
| Cattle | Dairy | Cows | Milking 1st half | 500 |  | 26 | 205.5 |
| Cattle | Dairy | Cows | Milking 1st half | 500 |  | 30 | 230.5 |
| Cattle | Dairy | Cows | Milking 2nd half | 500 |  | 5 | 65 |
| Cattle | Dairy | Cows | Milking 2nd half | 500 |  | 7 | 94.5 |
| Cattle | Dairy | Cows | Milking 2nd half | 500 |  | 9 | 109.5 |
| Cattle | Dairy | Cows | Dry or sterile | 400 |  | 0 | 66 |
| Cattle | Dairy | Cows | Dry or sterile | 500 |  | 0 | 78 |
| Cattle | Dairy | Cows | Dry or sterile | 400 |  | 0 | 78 |
| Cattle | Dairy | Cows | Dry or sterile | 500 |  | 0 | 86 |
| Cattle | Dairy | Bulls | Not coupling | 600 |  |  | 70 |
| Cattle | Dairy | Bulls | Not coupling | 700 |  |  | 78 |
| Cattle | Dairy | Bulls | Not coupling | 800 |  |  | 84 |
| Cattle | Dairy | Bulls | Not coupling | 900 |  |  | 91 |
| Cattle | Dairy | Bulls | Not coupling | 1000 |  |  | 97 |
| Cattle | Dairy | Bulls | Not coupling | 1100 |  |  | 102 |
| Cattle | Dairy | Bulls | Not coupling | 1200 |  |  | 108 |
| Cattle | Dairy | Bulls | Avg load | 600 |  |  | 76 |
| Cattle | Dairy | Bulls | Avg load | 700 |  |  | 83 |
| Cattle | Dairy | Bulls | Avg load | 800 |  |  | 90 |
| Cattle | Dairy | Bulls | Avg load | 900 |  |  | 97 |
| Cattle | Dairy | Bulls | Avg load | 1000 |  |  | 104 |
| Cattle | Dairy | Bulls | Avg load | 1100 |  |  | 110 |
| Cattle | Dairy | Bulls | Avg load | 1200 |  |  | 117 |
| Cattle | Dairy | Bulls | Inc load | 600 |  |  | 92 |
| Cattle | Dairy | Bulls | Inc load | 700 |  |  | 102 |
| Cattle | Dairy | Bulls | Inc load | 800 |  |  | 110 |
| Cattle | Dairy | Bulls | Inc load | 900 |  |  | 119 |
| Cattle | Dairy | Bulls | Inc load | 1000 |  |  | 127 |
| Cattle | Dairy | Bulls | Inc load | 1100 |  |  | 134 |
| Cattle | Dairy | Bulls | Inc load | 1200 |  |  | 141 |
| Sheep | Wool | Ewes | Preg 1st half | 50 |  |  | 12.5 |
| Sheep | Wool | Ewes | Preg 1st half | 60 |  |  | 13.5 |
| Sheep | Wool | Ewes | Preg 2nd half | 50 |  |  | 14.5 |
| Sheep | Wool | Ewes | Preg 2nd half | 60 |  |  | 16.5 |
| Sheep | Meat/wool | Ewes | Preg 1st half | 60 |  |  | 12.1 |
| Sheep | Meat/wool | Ewes | Preg 1st half | 70 |  |  | 13 |
| Sheep | Meat/wool | Ewes | Preg 2nd half | 60 |  |  | 16 |
| Sheep | Meat/wool | Ewes | Preg 2nd half | 70 |  |  | 17.2 |
| Sheep | Meat/tallow | Ewes | Preg 1st half | 60 |  |  | 13.5 |
| Sheep | Meat/tallow | Ewes | Preg 1st half | 70 |  |  | 14.5 |
| Sheep | Meat/tallow | Ewes | Preg 2nd half | 60 |  |  | 17.5 |
| Sheep | Meat/tallow | Ewes | Preg 2nd half | 70 |  |  | 18.5 |
| Sheep | Wool | Ewes | Suckling 1st half | 50 |  |  | 20 |
| Sheep | Wool | Ewes | Suckling 1st half | 60 |  |  | 23 |
| Sheep | Wool | Ewes | Suckling 2nd half | 50 |  |  | 15.5 |
| Sheep | Wool | Ewes | Suckling 2nd half | 60 |  |  | 17 |
| Sheep | Meat/wool | Ewes | Suckling 1st half | 60 |  |  | 22 |
| Sheep | Meat/wool | Ewes | Suckling 1st half | 70 |  |  | 23 |
| Sheep | Meat/wool | Ewes | Suckling 2nd half | 60 |  |  | 18.4 |
| Sheep | Meat/wool | Ewes | Suckling 2nd half | 70 |  |  | 19.2 |
| Sheep | Meat/tallow | Ewes | Suckling 1st half | 60 |  |  | 21 |
| Sheep | Meat/tallow | Ewes | Suckling 1st half | 70 |  |  | 22 |
| Sheep | Meat/tallow | Ewes | Suckling 2nd half | 60 |  |  | 18.5 |
| Sheep | Meat/tallow | Ewes | Suckling 2nd half | 70 |  |  | 19.5 |
| Sheep | Wool & meat/wool | Rams | Not coupling | 90 |  |  | 19 |
| Sheep | Wool & meat/wool | Rams | Not coupling | 100 |  |  | 20 |
| Sheep | Wool & meat/wool | Rams | Not coupling | 110 |  |  | 21 |
| Sheep | Meat/tallow | Rams | Not coupling | 80 |  |  | 19 |
| Sheep | Meat/tallow | Rams | Not coupling | 90 |  |  | 20 |
| Sheep | Meat/tallow | Rams | Not coupling | 100 |  |  | 21 |
| Sheep | Wool & meat/wool | Rams | Coupling | 90 |  |  | 24 |
| Sheep | Wool & meat/wool | Rams | Coupling | 100 |  |  | 25 |
| Sheep | Wool & meat/wool | Rams | Coupling | 110 |  |  | 26 |
| Sheep | Meat/tallow | Rams | Coupling | 80 |  |  | 24 |
| Sheep | Meat/tallow | Rams | Coupling | 90 |  |  | 25 |
| Sheep | Meat/tallow | Rams | Coupling | 100 |  |  | 26 |
| Sheep | Wool | Ewe lambs | 4-6 mos | 27.5 |  |  | 8.525 |
| Sheep | Wool | Ewe lambs | 6-8 mos | 33 |  |  | 9.625 |
| Sheep | Wool | Ewe lambs | 8-10 mos | 38 |  |  | 10.725 |
| Sheep | Wool | Ewe lambs | 10-12 mos | 41 |  |  | 11 |
| Sheep | Wool | Ewe lambs | 12-18 mos | 46 |  |  | 11.275 |
| Sheep | Meat/wool | Ewe lambs | 4-6 mos | 38.5 |  |  | 11 |
| Sheep | Meat/wool | Ewe lambs | 6-8 mos | 38.5 |  |  | 12.1 |
| Sheep | Meat/wool | Ewe lambs | 8-10 mos | 43 |  |  | 13.2 |
| Sheep | Meat/wool | Ewe lambs | 10-12 mos | 47.5 |  |  | 14.025 |
| Sheep | Meat/wool | Ewe lambs | 12-18 mos | 51.5 |  |  | 14.025 |
| Sheep | Wool | Ram lambs | 4-6 mos | 32 |  |  | 11.275 |
| Sheep | Wool | Ram lambs | 6-8 mos | 39.5 |  |  | 12.43 |
| Sheep | Wool | Ram lambs | 8-10 mos | 45 |  |  | 13.75 |
| Sheep | Wool | Ram lambs | 10-12 mos | 49.5 |  |  | 15.18 |
| Sheep | Wool | Ram lambs | 12-18 mos | 61.5 |  |  | 15.4 |
| Sheep | Meat/wool | Ram lambs | 4-6 mos | 36.5 |  |  | 13.2 |
| Sheep | Meat/wool | Ram lambs | 6-8 mos | 44.5 |  |  | 14.3 |
| Sheep | Meat/wool | Ram lambs | 8-10 mos | 52.5 |  |  | 15.4 |
| Sheep | Meat/wool | Ram lambs | 10-12 mos | 60 |  |  | 16.775 |
| Sheep | Meat/wool | Ram lambs | 12-18 mos | 70 |  |  | 17.325 |
| Horses | Meat | Stallions |  | 350 | 1000 |  | 93.2 |
| Horses | Meat | Stallions |  | 400 | 1000 |  | 97.4 |
| Horses | Meat | Stallions |  | 450 | 1000 |  | 101 |
| Horses | Meat | Stallions |  | 500 | 1000 |  | 108.8 |
| Horses | Meat | Stallions |  | 550 | 1000 |  | 112 |
| Horses | Meat | Stallions |  | 600 | 1000 |  | 122.4 |
| Horses | Meat | Foals | 0-1 mos | 70 | 1300 |  | 42 |
| Horses | Meat | Foals | 1-2 mos | 105 | 1000 |  | 45 |
| Horses | Meat | Foals | 2-3 mos | 133.5 | 900 |  | 47 |
| Horses | Meat | Foals | 3-4 mos | 160.5 | 900 |  | 52 |
| Horses | Meat | Foals | 4-5 mos | 187.5 | 900 |  | 56 |
| Horses | Meat | Foals | 5-6 mos | 214.5 | 900 |  | 60 |
| Horses | Meat | Foals | 6-7 mos | 235.5 | 900 |  | 60 |
| Horses | Meat | Foals | 7-8 mos | 258 | 1000 |  | 71 |
| Horses | Meat | Foals | 8-9 mos | 291 | 1300 |  | 88 |
| Horses | Milk | Mares |  |  | 400 | 10 | 84.8 |
| Horses | Milk | Mares |  |  | 400 | 12 | 92.1 |
| Horses | Milk | Mares |  |  | 400 | 14 | 98.4 |
| Horses | Milk | Mares |  |  | 500 | 14 | 105.7 |
| Horses | Milk | Mares |  |  | 500 | 16 | 113.1 |
| Horses | Milk | Mares |  |  | 500 | 18 | 120.4 |
| Horses | Milk | Mares |  |  | 500 | 20 | 126.5 |
