# Supplementary material for: Modeling the spatial distribution of grazing intensity in Kazakhstan
Source: PLoS One. 2019 Jan 11;14(1):e0210051. doi: 10.1371/journal.pone.0210051 (PMC6329506; doi:10.1371/journal.pone.0210051)
Supplement: S3 Table — Restrictions are noted when the age group is a subset of the Age/Sex in S2 Table. Nomenclature of the age group is as follows (square brackets enclose string variables): [*Beef/Dairy*][Function]_[*age*]_[*reproduction stage*]. *–if applicable. Function–common name considering age, sex, and castration. (DOCX) [file pone.0210051.s009.docx]

| Age group | Age/Sex in S2 Table | Period/weight restriction |
| --- | --- | --- |
| BeefCows | Beef Cows |  |
| BeefBulls_breeding | Beef Bulls |  |
| BeefHeifers | Beef Heifers |  |
| BeefHeifers_1to2yrs | Beef Heifers | 13-16 mos. |
| BeefHeifers_1to2yrs_inseminated | Beef Heifers | 17-20 mos. |
| BeefHeifers_2yrs | Beef Heifers | 17-20 mos. |
| BeefCalves_heifers_0to1yr | Beef Calves | 0-12 mos. |
| BeefCalves_bulls_0to1yr | Beef Calves | 0-12 mos. |
| BeefCalves_bulls_1yr | Beef Bull calves | 13-16 mos. |
| BeefSteers | Beef Bull calves |  |
| BeefOxen | Beef Bulls | Not coupling |
| BeefCattle_finishing | Beef Bull calves | 15-16 mos. |
| BeefBuffaloes | Beef Bulls | Not coupling |
| DairyCows | Dairy Cows |  |
| DairyBulls_breeding | Dairy Bulls |  |
| DairyHeifers | Beef Heifers |  |
| DairyHeifers_1to2yrs | Beef Heifers | 13-16 mos. |
| DairyHeifers_1to2yrs_inseminated | Beef Heifers | 17-20 mos. |
| DairyHeifers_2yrs | Beef Heifers | 17-20 mos. |
| DairyCalves_heifers_0to1yr | Beef Calves | 0-12 mos. |
| DairyCalves_bulls_0to1yr | Beef Calves | 0-12 mos. |
| DairyCalves_bulls_1yr | Beef Bull calves | 13-16 mos. |
| DairySteers | Beef Bull calves |  |
| DairyOxen | Beef Bulls | Not coupling |
| DairyCattle_finishing | Beef Bull calves | 15-16 mos. |
| DairyBuffaloes | Beef Bulls | Not coupling |
| EweDoe_1yr | Ewes |  |
| RamBuck_breeding | Rams |  |
| LambKid_0to1yr | Lambs |  |
| Wethers_1yr | Rams | Not coupling |
| Mares_3yrs | Mares |  |
| Stallions_breeding | Stallions |  |
| Foals_0to1yr | Foals |  |
| Fillies_1to3yrs | Stallions | 350 kg, 400 kg, 450 kg |
| Colts_1to3yrs | Stallions | 350 kg, 400 kg, 450 kg |
| Colts_3yrs | Stallions | 500 kg, 550 kg, 600 kg |
| Geldings | Stallions |  |
