# Supplementary material for: Modeling the spatial distribution of grazing intensity in Kazakhstan
Source: PLoS One. 2019 Jan 11;14(1):e0210051. doi: 10.1371/journal.pone.0210051 (PMC6329506; doi:10.1371/journal.pone.0210051)
Supplement: S4 Table — Production of each fodder type is recorded at the district level for each farm type. Consumption of each fodder type by each livestock species is recorded at the regional level (all farm types combined). The consumption of each fodder type by each livestock species at the district level for each farm type was estimated by multiplying the production of each fodder type by the consumption of each fodder type by each livestock species and dividing by the total consumption of each fodder type by each livestock species. (DOCX) [file pone.0210051.s010.docx]

| Feed class | ME (MJ)/kg Fod |
| --- | --- |
| FeedGrain | 12.98 |
| FeedLegume | 13.2 |
| SilageNonCorn | 1.98 |
| FeedRoot | 1.65 |
| FeedMelon | 0.88 |
| FeedCorn | 2.2 |
| CornGreenFodder | 2.2 |
| CornSilage | 2.2 |
| Hay | 5.72 |
| HayPasture | 5.72 |
| HayPastureCultivated | 5.72 |
| HayPastureSeeded | 5.72 |
| HayPastureNatural | 5.72 |
| HayAnnualGrass | 5.61 |
| HayPerennialGrass | 5.61 |
| GrassFodder | 2.75 |
| GrassFodderSeeded | 2.53 |
| GrassFodderNatural | 2.86 |
| GrassFodderAnnual | 1.87 |
| GrassFodderPerennial | 2.75 |
| SeedGrassAnnual | 3.41 |
| SeedGrassPerennial | 3.41 |
| GrazingGrassAnnual | 2.53 |
| GrazingGrassPerennial | 2.75 |
